# A phage repressor involved in the DNA-damage response of *Francisella*

**DOI:** 10.1093/pnasnexus/pgag114

**Published:** 2026-04-17

**Authors:** María Pérez-Varela, Miquel Sánchez-Osuna, Susana Campoy, Jesús Aranda, Jordi Barbé, Ivan Erill

**Affiliations:** Departament de Genètica i de Microbiologia, Facultat de Biociènces, Universitat Autònoma de Barcelona, Cerdanyola del Vallès, Barcelona 08193, Spain; Laboratori de Recerca en Microbiologia i Malalties Infeccioses, Hospital Universitari Parc Taulí, Institut d’Investigació i Innovació Parc Taulí (I3PT-CERCA), Universitat Autònoma de Barcelona, Sabadell, Barcelona 08208, Spain; Institut de Biotecnologia i Biomedicina, Universitat Autònoma de Barcelona, Cerdanyola del Vallès, Barcelona 08193, Spain; Departament de Genètica i de Microbiologia, Facultat de Biociènces, Universitat Autònoma de Barcelona, Cerdanyola del Vallès, Barcelona 08193, Spain; Departament de Genètica i de Microbiologia, Facultat de Biociènces, Universitat Autònoma de Barcelona, Cerdanyola del Vallès, Barcelona 08193, Spain; Professor Emeritus, Departament de Genètica i de Microbiologia, Facultat de Biociènces, Universitat Autònoma de Barcelona (UAB), Cerdanyola del Vallès (Barcelona), Spain; Departament d'Enginyeria de la Informació i de les Comunicacions, Universitat Autònoma de Barcelona, Cerdanyola del Vallès, Barcelona 08193, Spain; Department of Biological Sciences, University of Maryland Baltimore County, Baltimore, MD 21250, USA

## Abstract

Members of the S24 family of serine peptidases play central roles in bacterial and phage regulatory networks that respond to DNA damage. In most bacteria, the S24-family peptidase LexA functions as the master repressor of the SOS response, regulating the activity of genes involved in DNA repair and mutagenesis. LexA is known to be absent in some bacterial lineages, including genera of intracellular bacteria with substantial genomic reduction, but it has been shown that other S24-family peptidases can uptake its regulatory functions. In this work, we combine experimental and computational approaches to define the response to DNA damage in *Francisella*, a genus of facultative intracellular bacteria with moderate genome reduction that encodes a highly diverged LexA family protein. We use RNA sequencing to investigate the transcriptional response of *Francisella hispaniensis* to the DNA-damaging agent ciprofloxacin and reveal that canonical SOS genes are not induced upon DNA damage. Among the differentially expressed genes, we identify a gene encoding an S24-family peptidase that we term FddR (*Francisella* DNA-damage regulator). Electrophoretic mobility shift assays demonstrate that the product of this gene specifically binds to the palindromic sequence GTG-N_11_-CAC present in its promoter region, resulting in autoregulation. In silico analyses reveal that this S24-family peptidase is most likely a co-opted phage repressor that implements a DNA-damage response across the *Francisella* genus, regulating itself and divergently transcribed genes. Our results provide insights into the SOS repair system of the *Francisella* genus and its evolution, putting forward a mechanism for the gradual reconstruction of an SOS response network from self-contained divergently transcribed units.

Significance statement In all domains of life, cells must timely address DNA damage to remain viable. Serine peptidases of the S24 family are central regulators of the DNA-damage response across bacteria. However, the role of divergent members of the LexA protein family in the SOS response of different bacterial clades remains poorly understood. In this work, we demonstrate that *Francisella* species use a highly diverged member of the LexA family, *Francisella* DNA-damage regulator (FddR), to orchestrate their DNA-damage response. FddR, which we show was likely co-opted from a bacteriophage, autoregulates its own expression and controls divergently transcribed genes, forming a compact regulatory network. Our findings illustrate how broadly divergent members of the S24 peptidase family can be repurposed to implement SOS-like networks in intracellular bacteria with reduced genomes, providing new insights into the evolution and adaptability of DNA repair regulatory systems.

## Introduction

S24-family peptidases are key elements in the decision-making process of both bacteria and phages. These proteins, members of the serine protease superfamily, contain an N-terminal helix-turn-helix (HTH) DNA-binding domain and a C-terminal serine protease domain. The protease domain can selectively cleave alanyl bonds of the protein and disrupt the DNA-binding domain (1). S24-family peptidases operate primarily as transcriptional repressors and share a conserved mechanism to trigger their self-catalytic cleavage in response to DNA damage (1). This process is mediated by the RecA protein, which binds single-stranded DNA resulting from DNA damage and forms nucleoprotein filaments that promote the self-cleavage of S24-family peptidases (2, 3).

In bacteria, S24-family peptidases are exemplified by the LexA repressor, which orchestrates the SOS response against DNA damage by directly regulating the expression of multiple genes involved in DNA repair, cell-cycle control, and translesion DNA synthesis (4, 5). LexA binds to a specific sequence located upstream of target genes, known as the SOS box, which differs among bacterial groups and often even within the same phylogenetic group (4). In phages, S24-family peptidases are primarily represented by the CI (lambda repressor encoded by the *cI* gene) of lambdoid phages. This repressor, together with the Cro repressor, implements a well-studied bistable regulatory circuit that determines whether the phage enters the lytic or lysogenic cycle. In this system, the self-catalytic cleavage of CI in the face of DNA damage triggers the onset of the lytic cycle (6).

The LexA protein is widespread across the domain bacteria, but it is often absent in bacterial species that present a significant reduction in genome size and in the number of encoded genes (4, 7). This encompasses strict intracellular bacteria, such as *Chlamydia*, *Rickettsia*, and *Coxiella* (8, 9) and, to a lesser extent, facultative intracellular bacteria, such as *Brucella* and *Listeria*, which can live both inside and outside host cells and present moderate genome reduction (10).

S24-family peptidases often cohabit in a single bacterial genome, whether as a result of gene duplication or lateral gene transfer via domestication of S24-family peptidase-harboring mobile genetic elements (11, 12). Following the loss of the LexA repressor orchestrating the SOS response, other S24-family peptidases may take up its role and implement an SOS response through the gradual recruitment of canonical SOS genes. This process is facilitated by the mechanism for RecA-mediated self-catalytic cleavage that is conserved among S24-family peptidases (1). SOS networks have been reported under control of secondary bacterial and phage S24-family peptidases (12–15), but the evolutionary processes that support the convergent evolution of SOS networks under control of heterologous S24-family peptidases are not well understood.

*Francisella* is a genus of facultative intracellular bacteria with moderate genomic reduction in comparison with other intracellular pathogens (16). The most studied species within this genus is *Francisella tularensis*, the causative agent of the zoonotic disease tularemia. In the early 2000s, a new species of pathogenic *Francisella* was described in Spain and named *Francisella hispaniensis* (17). The genomes of *F. tularensis* and other *Francisella* species (18, 19) reveal that this genus possesses a full component of the DNA repair and translesion synthesis genes that are typically members of the SOS response in other clades.

In this work, we leverage transcriptomic data for *F. tularensis* and *F. hispaniensis* following ciprofloxacin treatment to analyze the response to DNA damage in the *Francisella* genus. We show that canonical SOS genes are not induced in response to ciprofloxacin in *Francisella*, but we identify a small subset of genes that are strongly induced in both *Francisella* species. One of these genes is annotated as encoding a LexA family protein mapping to COG1974 (20). We show that this protein, here termed FddR (*Francisella* DNA-damage regulator), self-regulates its own expression by binding to a palindromic DNA-binding motif. Comparative genomics analyses indicate that this regulator is conserved across the *Francisella* genus, where it is systematically found in divergent transcriptional units with other genes. These FddR-regulated genes include translesion synthesis DNA polymerases, which are often seen as the first component of DNA-damage response systems (4, 21). This puts forward a mechanism by which a transcriptional regulatory network may be assembled through the gradual accumulation of divergent transcriptional units associated with a regulator.

## Results

### Transcriptomic response to DNA damage in *Francisella* species

**Figure 1:**
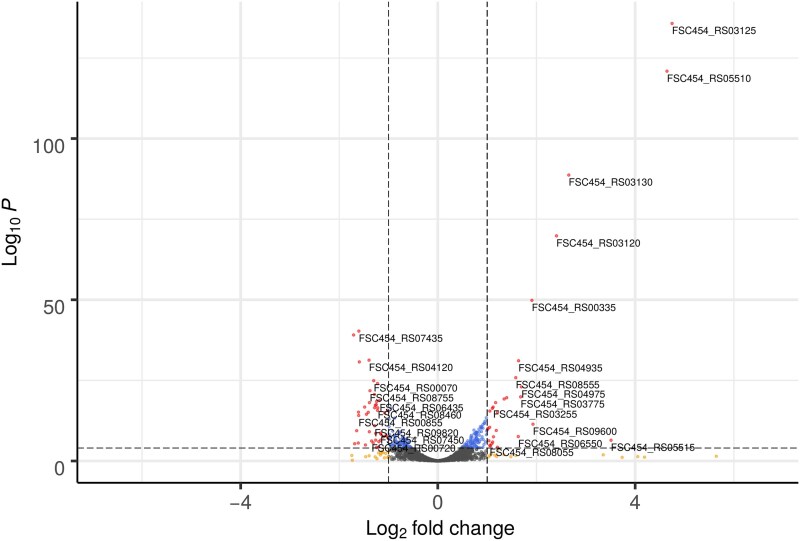
Volcano plot of differentially expressed genes between the ciprofloxacin-treated and the control cultures. Significantly differentially expressed genes are highlighted in red (log_2_-fold change ≥±1.0 and adjusted *P*-value of <0.05). Nonsignificantly differentially expressed genes are shown in either yellow (log_2_-fold change within ±1.0 without statistical significance), blue (statistically significant but with no log_2_-fold change differences), or gray (not meeting either threshold).

To study the response to DNA damage of *Francisella* species, we first studied the differences between the transcriptional profiles of ciprofloxacin-treated (0.125 mg/L) and control *F. hispaniensis* cultures. Genes exhibiting a log_2_-fold change value >1.0 or <1.0 and with a *P*-value <0.01 were considered differentially expressed genes. From the comparison between the control culture and the ciprofloxacin-treated culture, the number of genes with changes in their expression was 88 (Fig. 1; Table S1). Of these, 37 genes were up-regulated, indicating an increase in their expression in the presence of ciprofloxacin, and 51 genes were found to be down-regulated under such conditions. Several genes related to oxidation-reduction reactions as well as membrane transporters showed alterations in their expression levels but the only canonical SOS gene with significant change in expression was *ruvA*, encoding the Holliday junction branch migration complex subunit A, which was down-regulated. All the other canonical SOS genes detected in *F. hispaniensis* showed differences in basal expression levels but no significant changes in expression in response to ciprofloxacin (Fig. S1).

**Figure 2:**
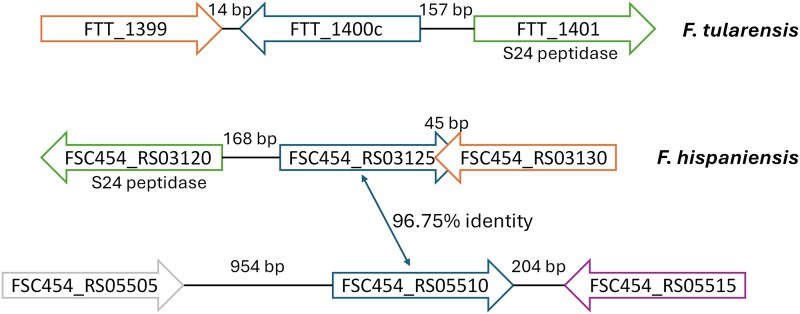
Diagram of the genetic neighborhood of genes up-regulated in response to ciprofloxacin treatment in both *F. hispaniensis* and *F. tularensis*, and their paralogs in *F. hispaniensis*.

We contextualized these results in *F. hispaniensis* by leveraging available RNA-seq data from *F. tularensis* exposed to an inhibitory ciprofloxacin concentration, which reveals significant up-regulation of 136 genes and down-regulation of 98 genes (22). In both studies, bacteria were grown in rich media appropriate for their specific nutritional requirements (brain heart infusion [BHI] supplemented with 1% glucose and 0.1% cysteine for *F. hispaniensis* and cation-adjusted Muller–Hinton broth, supplemented with 2% IsoVitaleX and 3 μM hematin, for *F. tularensis*). Bacteria were subsequently incubated with 0.5× minimum inhibitory concentration (MIC) ciprofloxacin for 2 h. When comparing both ciprofloxacin-induced transcriptomes, only three genes (FSC454_RS03120, FSC454_RS03125, and FSC454_RS03130) showed significant differential expression across both datasets. These three genes map to a region with an identical genetic arrangement in both genomes and constitute the conserved transcriptomic response to ciprofloxacin treatment in these two *Francisella* species (Fig. 2). FSC454_RS03125 is annotated as a normocyte-binding protein 2, although no homology with any known domains or structures can be identified with HHpred (23), and FSC454_RS03130 is annotated as a DMT family transporter. The *F. hispaniensis* genome also contains a paralog of FSC454_RS03125 (96.75% sequence identity; Fig. 2). This paralog (FSC454_RS05510) is annotated as a hypothetical protein and is also strongly up-regulated (4.64 log_2_-fold) in response to ciprofloxacin, together with the adjacent FSC454_RS05515 (deoxy-nucleoside triphosphate pyrophosphohydrolase; 3.51 log_2_-fold; Fig. 2). Both paralogs have a convergent, head-to-head genetic arrangement with their adjacent up-regulated genes, suggesting that the detected up-regulation in the adjacent genes might be the result of transcriptional read through. Inspection of the RNA-seq read distribution on these two genomic loci revealed that reads for FSC454_RS03130 and FSC454_RS05515 peaked on their 3′ ends, supporting the hypothesis of transcriptional read through, which was subsequently confirmed by RT-PCR (Fig. S2).

The remaining up-regulated gene, FSC454_RS03120, encodes a LexA family protein that contains an HTH (PF01381) domain, and the peptidase S24-like (PF00717) domain involved in RecA-mediated self-catalytic cleavage. We validated the up-regulation of the FSC454_RS03120 gene in response to ciprofloxacin treatment using RT-qPCR. The results of the RT-qPCR assays following ciprofloxacin treatment showed consistent induction of the expression of this S24-family peptidase-encoding gene (3.6-, 6.9-, and 7.6-fold across three biological replicates), corroborating the RNA-seq results and suggesting a potential autoregulatory mechanism. Given that the RecA recombinase has a well-established role as the activator of the SOS response, we also assessed by RT-qPCR the expression levels following ciprofloxacin treatment of the gene annotated as encoding RecA (FSC454_RS00525). However, and in agreement with the RNA-seq data, no significant changes in expression levels (0.68 and 1.02 on two biological replicates) were observed.

### Phylogeny of *Francisella* S24-family peptidase regulators

**Figure 3:**
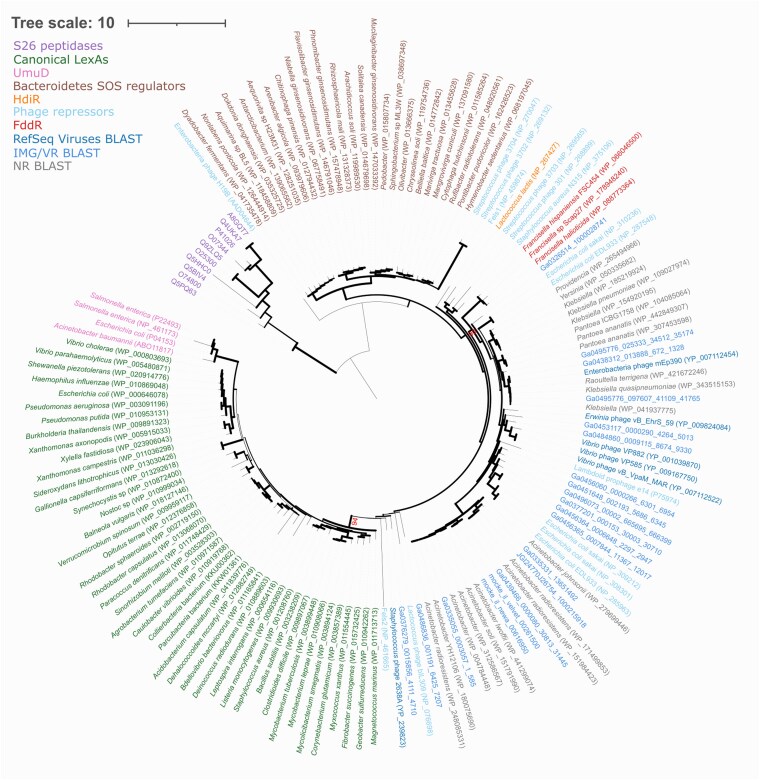
Rooted phylogenetic tree of S24-family peptidase family protein sequences. FddR homologs were combined with S24-family sequences from a previous study (15). To broaden the phylogenetic context and incorporate phage-associated homologs, additional sequences were retrieved by BLASTP searches using FddR (FSC454_RS03120) as the query against the NCBI nonredundant database (excluding Francisellaceae), as well as the NCBI RefSeq Viruses and IMG/VR v4 databases. S26 peptidase representative sequences from the IPR015927 superfamily were included as the outgroup. Relevant bootstrap support values were included as percentage, and the rest were represented as branch width. A rectangular and complete version of this phylogenetic tree is included in Fig. S3.

The up-regulation of a gene encoding a putative S24-family peptidase regulator following ciprofloxacin treatment in *F. hispaniensis* and *F. tularensis* prompted us to investigate to what extent its product might regulate an SOS response in *Francisella* species. To determine whether other members of this genus encode homologs of this S24-family peptidase, we performed reciprocal BLASTP searches with the FSC454_RS03120 protein sequence against the proteomes encoded by all the *Francisella* complete genomes available in GenBank (*n* = 118). This analysis identified orthologs for the S24-family peptidase regulator in 97.6% of all available *Francisella* complete assemblies (Table S2). To elucidate the relationship between these putative regulators and other S24-family members known to regulate SOS networks, we inferred their phylogeny using a protein multiple sequence alignment with representative members of COG1974 and COG2932 (15). COG1974 encompasses several homologs of the *Escherichia coli* UmuD protein and a well-defined group of LexA proteins, termed canonical LexA proteins, that includes the *E. coli* and *Bacillus subtilis* LexA repressors. COG2932 encompasses phage repressors and the bacterial HdiR repressor (15). The resulting tree (Figs. 3 and S3; Data S1 and S2) showed that the S24-family peptidases from *Francisella* do not cluster with canonical LexAs, UmuD proteins or the recently characterized Bacteroidetes SOS regulators. Rather, they define a well-supported clade (90% bootstrap support) with lambdoid phage repressors.

**Figure 4:**
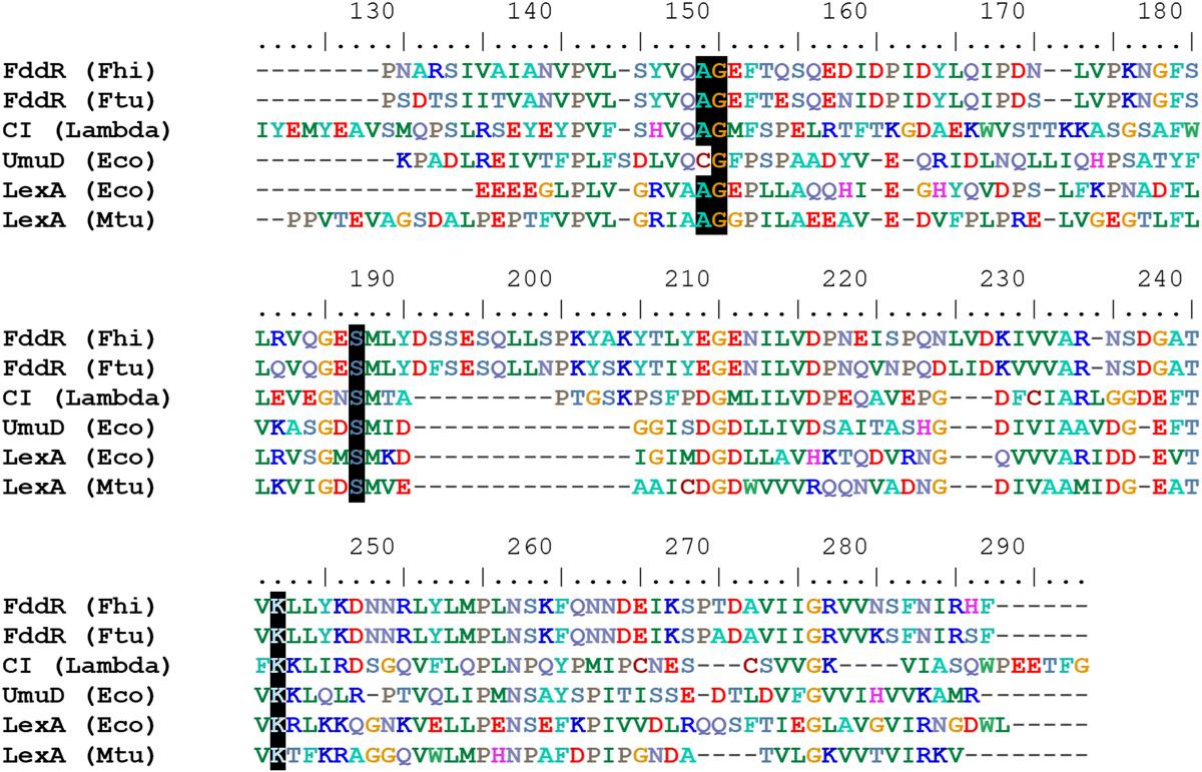
Multiple sequence alignment including the C-terminal domain of the *F. hispaniensis* (Fhi) and *F. tularensis* (Ftu) FddR S24-family peptidases and reference S24 proteins: *E. coli* (Eco) LexA and UmuD, *Mycobacterium tuberculosis* (Mtu) LexA and the Lambda phage CI repressor. The conserved Ser–Lys catalytic dyad and Ala–Gly peptide bond residues are highlighted.

These include experimentally characterized lambdoid phage repressors, such as the CohE repressor of *E. coli* prophage e14 (24), as well as putative repressors from known lambdoid prophages, such as the Sp10 prophage in *E. coli* O157:H7 str. Sakai (25), and multiple sequences from the IMG/VR and RefSeq Viruses databases. This suggests that the *Francisella* S24-family peptidases derive from lambdoid prophage repressors, rather than from canonical LexA proteins, which conform their own, well-supported (94% bootstrap support) clade (Fig. 3). Despite their association with lambdoid phage repressors, the *Francisella* genes encoding these S24-family peptidases do not overlap with prophages predicted by PhiSpy in their respective genomes (26) (Table S3). This is in contrast with the closest homologs of *Francisella* S24-family peptidases detected in other Gammaproteobacteria species, which map to the *Francisella* FddR clade in Fig. 3. These proteins, such as WP_265494966 from *Providencia* or WP_442849307 from *Pantoea ananatis*, have low amino acid sequence identity (<35%) with *Francisella* S24-family peptidases and are frequently encoded in genomic regions mapping to PhiSpy-predicted prophages (Table S3). Together with the widespread distribution and high-sequence identity of these S24-family peptidases across *Francisella*, these data suggest that *Francisella* S24-family peptidases were likely acquired in an ancestor of this genus. S24-family proteins undergo self-catalytic cleavage mediated by RecA nucleoprotein filaments through the interaction of a Ser–Lys catalytic dyad with a cleavage site defined by an Ala–Gly peptide bond (27). A multiple sequence alignment with reference proteins from the S24 family revealed that both these elements are conserved in *Francisella* S24-family peptidase regulators (Fig. 4). Taken together, these data suggest that these S24-family peptidase regulators define a novel protein family involved in the DNA-damage response of the *Francisella* genus, and we hereafter refer to these S24-family peptidase regulators as FddR.

### FddR regulates its own transcription by binding to its upstream promoter region

**Figure 5:**
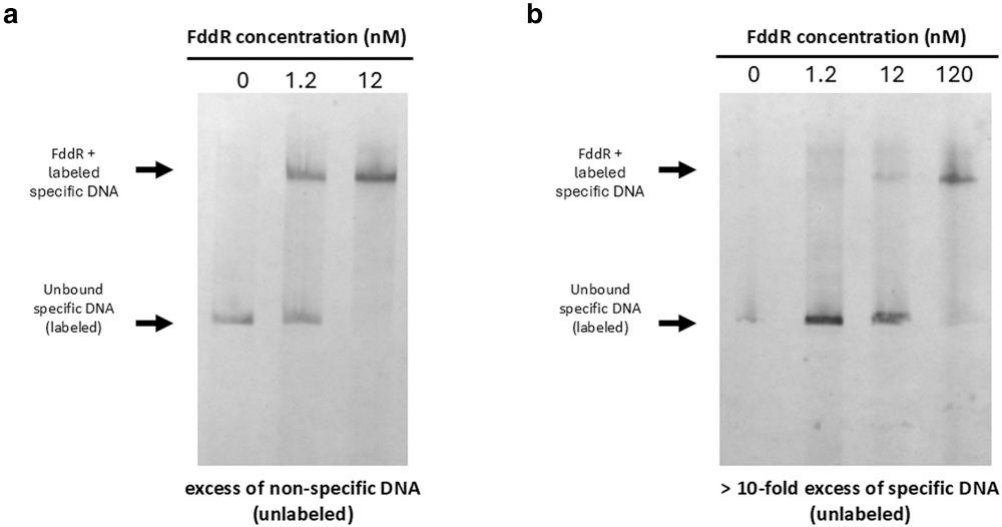
a) Electrophoretic mobility of a DIG-labeled DNA probe containing the upstream promoter region of the FSC454_RS03120 gene of *F. hispaniensis*. b) Electrophoretic mobility in the presence of increasing concentrations of the purified FddR protein with an excess of nonspecific or specific unlabeled DNA.

The RNA-seq and phylogenetic inference results suggested that FddR could be a noncanonical regulator of the DNA-damage response in *F. hispaniensis*. To determine the ability of this S24-family peptidase to regulate gene expression by binding to DNA, we conducted electrophoretic mobility shift assays (EMSAs) using purified *F. hispaniensis* FddR protein (WP_066046500) and the upstream promoter region of the *fddR* gene (FSC454_RS03120). A mobility shift indicative of DNA–protein interaction was observed when the purified protein was added to a DNA fragment containing the upstream region of the *fddR* gene (Fig. 5a). The specificity of this interaction was confirmed through the addition of an excess of unlabeled promoter DNA as a competitive control, which abolished the interaction (Fig. 5b). These data indicate that the *F. hispaniensis* FddR protein binds specifically to a sequence within the promoter region of the *fddR* gene. Given that RNA-seq and RT-qPCR results show that the *fddR* gene is strongly induced in response to ciprofloxacin treatment, our results indicate that the FddR regulates its own transcription through specific binding. This autoregulatory mechanism is consistent with the known behavior of S24 regulators in both bacteria and phages, where they play a critical role in controlling the SOS response to DNA damage and the lysis–lysogeny switch (4, 6, 28).

### FddR targets a palindromic motif

**Figure 6:**
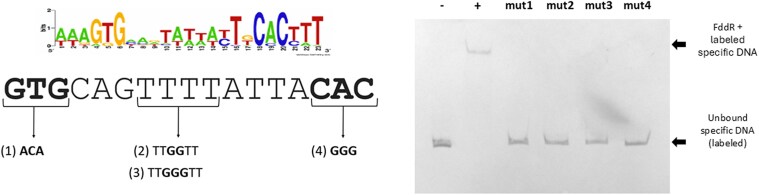
Electrophoretic mobility of DIG-labeled DNA probes encompassing variants of the predicted FddR-binding site within the fddR promoter in the presence of purified FddR protein (12 mM). The FddR-binding motif inferred by MEME is shown as a sequence logo. Modifications to the FddR-binding site within the fddR promoter are indicated by arrows and numbers. The mobility of the wild-type DNA fragment in the absence (−) or presence (+) of the same amount of purified FddR protein is shown as a control.

To characterize the specific region recognized by the FddR and identify the precise nucleotides involved in binding, we performed motif inference on a comparative genomics analysis dataset. We obtained the nucleotide sequence upstream of FddR homologs from all *Francisella* complete assemblies on GenBank and used multiple EM for motif elicitation (MEME) to detect overrepresented motifs. As shown in Fig. 6, MEME identified a significant motif with palindromic structure (GTG-N_11_-CAC) in the upstream regions of these FddR homologs. To further characterize this motif, we performed site-directed mutagenesis on a sequence (aGTGcagttttattaCACc) within the *fddR* promoter region matching this motif. We observed that modifications to any of the dyad nucleotides resulted in an abolition of the observed gel retardation (Fig. 6). The same results were observed when changes in spacer length were introduced, with a one-base and three-base insertion in the middle of the TTTT central motif effectively abolishing binding, but not when substitutions were introduced in the spacer region (Figs. 6 and S4). Together, these findings demonstrate that the *F. hispaniensis* putative SOS regulator binds specifically to a palindromic GTG-N_11_-CAC motif.

### FddR homologs define an SOS-like regulatory network in the *Francisella* genus

**Figure 7:**
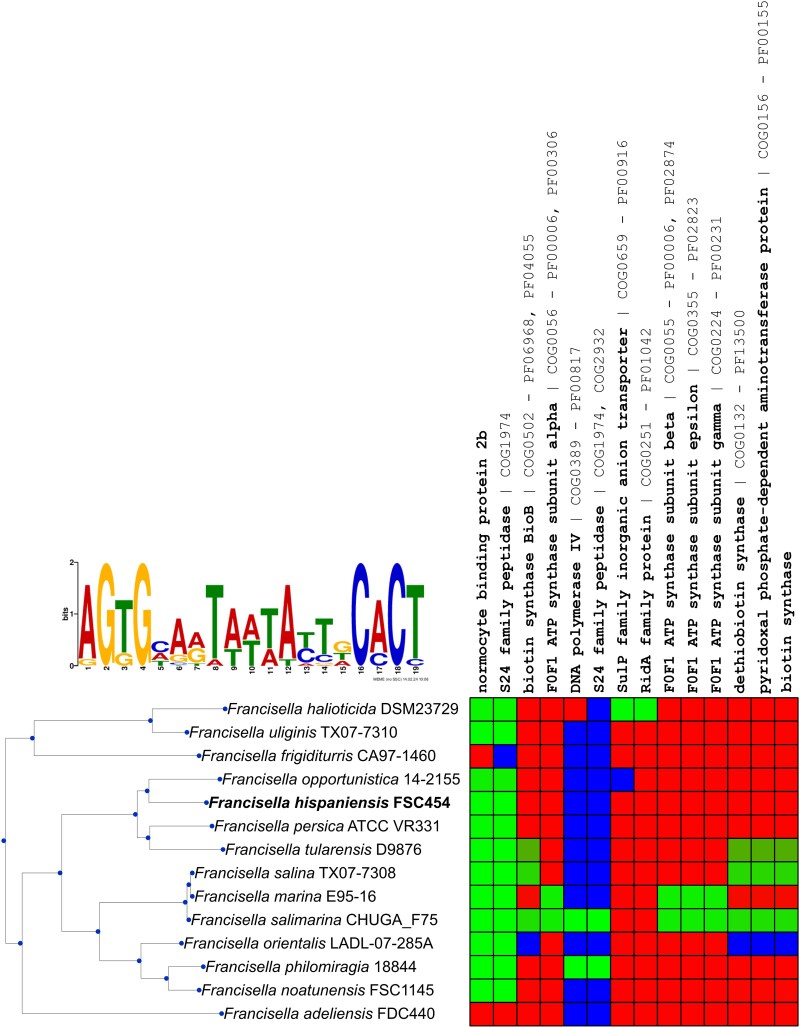
Comparative analysis of the FddR regulon. The MEME-inferred FddR-binding motif used to search for sites is shown as a sequence logo. CGB-generated heat map depicting the FddR regulon across 14 complete *Francisella* genomes. Each column represents an orthologous group, sorted by average posterior probability of regulation and labeled with *F. hispaniensis* annotations and CGB-assigned COG and PFAM identifiers. Cells are colored from green (regulation) to red (no regulation); blue denotes ortholog absence.

**Figure 8:**
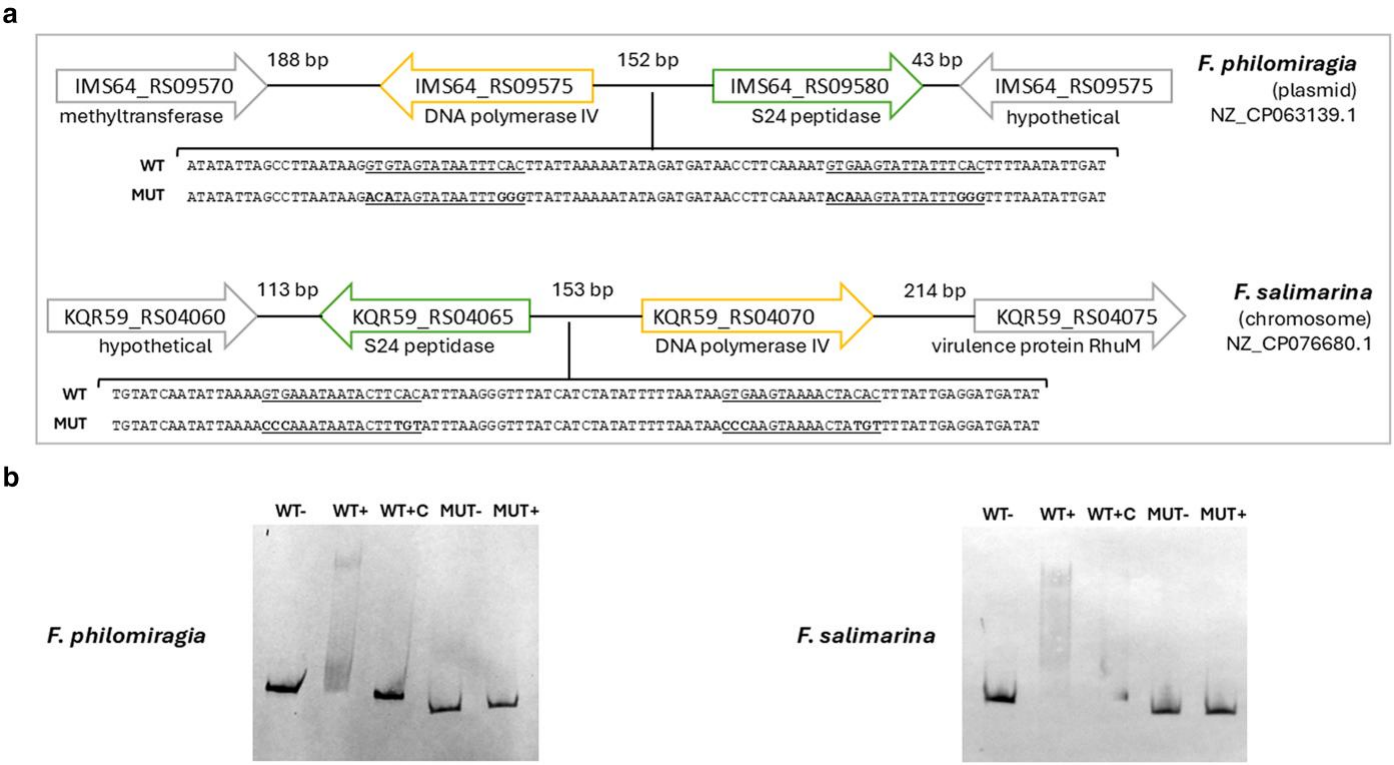
a) Schematic diagram of the genetic neighborhood of genes encoding FddR paralogs in *F. philomiragia* and *F. salimarina*. Wild-type and mutant probes are shown, the palindromic motif is underlined, and substituted nucleotides are in bold. b) Electrophoretic mobility of DIG-labeled DNA fragments containing the dinB upstream region from either *F. philomiragia* or *F. salimarina* in the presence of purified *F. hispaniensis* FddR (12 mM). The (−) symbol indicates absence of protein and the (+) symbol indicates presence of the purified S24-family peptidase. The (+C) symbol denotes the presence of unlabeled competitor DNA. WT and MUT indicate whether the probe is the wild-type probe or the mutant probe containing the substituted nucleotides shown in (a).

Given the DNA-binding activity of FddR, we investigated whether this protein controls a regulon potentially involved in DNA repair using a comparative genomics approach. We used the MEME-inferred FddR-binding motif as input for the comparative genomics browser (CGB) and performed an analysis of the putative regulatory network in reference RefSeq *Francisella* genomes. The results presented in Fig. 7 reveal a small regulatory network dominated by FddR and the divergently transcribed gene associated with it. This genomic locus appears to be highly conserved and systematically regulated by the FddR-binding motif within the *Francisella* genus, defining the core DNA-damage response coordinated by FddR in *Francisella*. This core response network is complemented in two *Francisella* species (*Francisella philomiragia* and *Francisella salimarina*) by another set of two genes with divergent orientation, encoding an FddR paralog and a DinB DNA polymerase IV (Figs. 7 and 8). The proteins encoded by these two *fddR-dinB* loci show significant sequence identity (>95%) and a preserved divergent organization, even though they have markedly different genomic environments and are harbored by different molecules (an unnamed plasmid in *F. philomiragia* and chromosomal DNA in *F. salimarina*; Fig. 8). To assess whether FddR binds to the predicted FddR-binding-site upstream of the *dinB* gene in both *F. philomiragia* and *F. salimarina*, we conducted EMSAs using the purified *F. hispaniensis* FddR protein and probes containing the predicted FddR-binding sites and mutant variants. The EMSA results revealed that *F. hispaniensis* FddR binds to the region upstream of the *dinB* genes of *F. philomiragia* and *F. salimarina* and that it does so specifically by targeting the predicted FddR-binding sites (Fig. 8).

The FddR paralogs in *F. philomiragia* and *F. salimarina* show ∼60% sequence identity with the bona fide orthologs of the *F. hispaniensis* FddR protein in these two species. To determine whether these divergent proteins target the same binding motif as the *F. hispaniensis* FddR protein, we performed reciprocal EMSAs using the *F. hispaniensis* FddR and its purified *F. salimarina* paralog on probes containing the upstream regions of the *F. hispaniensis fddR* and the *F. salimarina dinB* genes. Our results (Figs. 8 and S5) show that both FddR proteins bind the predicted FddR-binding sites in both species (Figs. 8 and S5), indicating that the paralogous FddR proteins recognize the same motif as the *F. hispaniensis* FddR protein. Taken together, these findings indicate that FddR homologs define a consistent DNA-damage response network in *Francisella* via regulatory modules comprising an *fddR* gene together with a divergently transcribed gene that may encode either uncharacterized proteins or the canonical SOS response member DinB.

## Discussion

In this work, we combined experimental and computational approaches to elucidate the DNA-damage response of *Francisella* species. RNA-seq analysis of ciprofloxacin-treated and control *F. hispaniensis* cultures revealed 88 genes with altered expression, and no homologs of canonical SOS genes were shown to be up-regulated (Fig. 1). Furthermore, comparison with similar data from *F. tularensis* identified only three genes that are significantly up-regulated in response to ciprofloxacin in both species, suggesting that they conform the core response to DNA damage in this genus (Fig. 2). These genes included an S24-family peptidase that is consistently present in available *Francisella* genomes. This peptidase belongs to the same family as LexA and phage repressors, which undergo RecA-mediated self-catalytic cleavage in the presence of DNA damage. A phylogenetic analysis revealed that this S24-family peptidase, here termed FddR, defines a well-supported clade with lambdoid phage repressors and contains all the key residues required to operate as a DNA-damage response regulator (Figs. 3 and 4).

Combining comparative genomics and experimental approaches, we defined the FddR-binding motif (GTG-N_11_-CAC), which has a palindromic structure that is characteristic of S24-family peptidase regulators (4) and which enabled us to analyze in silico the FddR regulatory network across the *Francisella* genus (Fig. 5). Our results reveal a small but highly conserved network defined by *fddR* and an associated divergently transcribed gene of unknown function. This core network is complemented in some species, like *F. salimarina*, by an *fddR* paralog and a divergently transcribed *dinB* gene (Figs. 7 and 8). The absence of a LexA repressor orchestrating a functional SOS response has been documented in intracellular pathogens like *Rickettsia*, *Coxiella*, and *Mycoplasma* (29–31). This loss has been attributed to the substantial genomic reduction these genera have undergone and to a possible need for these pathogens to maintain constitutive expression of DNA repair genes in the adverse environment of the host cell (4).

*Francisella* species are also facultative intracellular pathogens with significant genomic reduction and, therefore, the absence of a canonical LexA repressor may have been adaptive in this genus. It has been argued that intracellular pathogens compensate for the loss of a functional SOS response through constitutive expression of nucleotide excision and recombination pathways (29, 30, 32), and our findings reveal that elements of these pathways, such as the *recA* and *uvrA* genes have high levels of basal expression (Fig. S1). A functional SOS response has been shown to be instrumental in the survival of bacteria to ciprofloxacin treatments, both through the induction of mutations in the genes encoding DNA gyrase and topoisomerase IV, and through the induction of persistence (33–35). The product of the gene that is primarily regulated by FddR in *Francisella* species (the *F. hispaniensis* FSC454_RS03125 gene) has no known function, but it could conceivably participate in increasing the mutation rate. The fact that homologs of DinB, an error-prone polymerase, are regulated by FddR in some *Francisella* species supports the hypothesis that this phage repressor has been co-opted by the cell to orchestrate induced mutagenesis in response to ciprofloxacin and other DNA-damaging agents.

The SOS response to ciprofloxacin is triggered by double-stranded DNA breaks and stalled replication forks in a RecA-dependent manner (33). Our results show that the conserved residues necessary for the RecA-mediated self-catalytic of S24-family peptidases are conserved in FddR sequences (Fig. 4), indicating that induction of FddR in response to ciprofloxacin is most likely mediated by RecA, despite the fact that the *recA* gene is not induced by ciprofloxacin treatment. While RecA is typically a member of the SOS regulatory network, functional SOS networks in which the *recA* gene is not regulated by LexA have been documented before (36–39).

In free-living bacteria, like the Bacteroidetes, large SOS response networks have been documented to be under control of phage-like repressors, suggesting that a captured phage repressor may reconstitute a functional SOS network following the loss of the canonical *lexA* gene (15). Global regulatory networks, like the SOS response, evolve through the expansion of local regulatory networks, in which the regulator gene typically forms an operon or a divergently transcribed unit with the regulated genes (40). The self-contained nature of these regulatory units, which have been described for LexA and other S24-family peptidase regulators (4, 15), also facilitates their dissemination through lateral gene transfer, enabling the convergent evolution of regulatory networks (40).

In this context, the discovery of a phage repressor controlling a small DNA-damage response network in *Francisella* provides some insights into the possible evolutionary tradeoffs and paths straddling the line between complete loss and full reconstruction of the SOS response. FddR homologs are widespread across *Francisella*, but absent outside this genus, suggesting that they were acquired by an ancestor of extant *Francisella* species and that they have since been retained to implement a basic DNA-damage response. Our work also reveals that instances of FddR presenting substantial sequence divergence coexist in some *Francisella* genomes, arranged in divergently transcribed units with different genes. The fact that these diverged FddR proteins still target the same DNA-binding motif suggests that regulation of a single functional gene may be sufficient to prevent divergence of the FddR-binding motif (13). This provides an evolutionary mechanism for the initial reconstruction of a DNA-damage response under the control of a phage-like regulator. *Francisella* cells may gradually acquire FddR regulatory modules harboring different regulated genes, and subsequently lose the redundant FddR proteins, eventually giving rise to more complex SOS-like regulatory networks such as the one seen in the Bacteroidetes.

## Materials and methods

### Strains and growth conditions

*Escherichia coli* (DHα or BL21) cells were grown in Lysogeny Broth (LB) medium and when necessary, the medium was supplemented with ampicillin (50 mg/L), chloramphenicol (34 mg/L) or X-gal (40 mg/L). *Francisella hispaniensis* FSC454 (DSM 22475) was grown in BHI media supplemented with 1% glucose and 0.1% cysteine and incubated at 37 °C with shaking at 180 rpm. For ciprofloxacin treatment, an overnight culture of the wild-type strain was grown and diluted 1:100 in fresh medium on the following day. Cultures were grown to exponential growth phase and then treated with ciprofloxacin (0.125 mg/L) for 2 h.

### Total RNA extraction and RT-qPCR

*Francisella hispaniensis* strain DSM 22475 was grown in BHI media supplemented with 1% glucose and 0.1% cysteine and incubated overnight. A 1:100 dilution in fresh medium was performed, and cells were grown to exponential growth phase. Cultures were divided into a control culture without ciprofloxacin and a treatment culture with ciprofloxacin and incubated at 37 °C with shaking for 2 h. Cells were harvested by centrifugation at 8,000 rpm for 10 min at 4 °C, and pellets were frozen at −80 °C. For cell lysis, pellets were resuspended in Tris-ethylene diamine tetra-acetic acid (EDTA) buffer and treated with lysozyme (50 mg/mL) for 10 min at 37 °C vortexing every 2 min. Total RNA was extracted using the RNeasy Kit (Qiagen) and following the manufacturer's protocol. DNA depletion was carried out using the DNase Turbo Kit (Ambion) and the absence of DNA was confirmed by PCR.

Gene expression was determined by RT-qPCR using the Lightcycler RNA Master SYBR green I (Roche) on a Lightcycler 480 instrument (LC480, Roche) following the manufacturer's instructions. RNA concentrations were adjusted, and specific oligonucleotides were generated to amplify a fragment of ∼200 bp of the genes of interest (Table S4). Expression levels of the target genes were standardized relative to the *tul4* gene using the threshold cycle (2^−ΔΔCT^) method (41), and three biological replicates were carried out for each experiment.

### RNA-seq and analysis

For RNA extraction, control and ciprofloxacin-treated cultures were grown, and RNA isolated from each as described above. Three independent sets of RNA were obtained, and transcriptomic analysis was performed by *Servei de Genòmica-UAB* (Barcelona, Spain). Ribosomal RNA was removed using the Ribo-Zero Plus rRNA Depletion Kit (Illumina), and cDNA libraries were obtained according to Illumina's recommendations. The cDNA fragments were ligated to adaptors, and then the products were purified and enriched by PCR. Library quality was assessed on a Bioanalyzer (Agilent DNA 1000 Chip). The library set was sequenced with MiSeq Run sequencer (Illumina).

RNA-seq analysis was carried out by the Multi-omics Bioinformatics Core facility-UAB (Barcelona, Spain). The raw sequencing data were checked for quality, trimmed, and then mapped to the *F. hispaniensis* FSC454 reference genome (NZ_CP018093) using Hisat2 RNA aligner. Quantification of feature counts was performed using FeatureCounts for the RefSeq annotation and then, normalization and differential expression of gene counts was analyzed using DESeq2. The expression of a gene was considered induced or repressed when the fold change of the treated culture compared to the control culture was ≥2 or ≤−2, respectively, and the *P*-value of the difference compared with the control was <0.01. Data have been deposited to the Gene Expression Omnibus database under the accession number: GSE308416.

### FddR protein purification

The FddR protein encoding gene (FSC454_RS03120) was amplified by PCR using the primers Fh FSC454_RS03120 XhoI F 5′-ATCGCTCGAGATGTTTGACTAC-3′ and Fh FSC454_RS03120 BamHI R 5′-AGGGATCCTTAGAAGTGTCTG-3′. The purified PCR fragment was digested with the restriction enzymes XhoI and BamHI and cloned in the multicloning site of the pET15b vector (Millipore) digested with the same restriction enzymes and dephosphorylated. Ligation was introduced into *E. coli* DH5α cells by electroporation and transformants selected on LB plates with ampicillin and chloramphenicol. Correct constructs were confirmed by PCR and sequencing (Macrogen) using the universal T7 Promoter and T7 Terminator primers and then introduced into *E. coli* BL21-CodonPlus (DE3)-Ril (Stratagene) competent cells for overexpression. An overnight culture of *E. coli* BL21 containing the pET15b + FSC454_RS03120 vector was grown in LB with ampicillin, chloramphenicol, and 1% glucose. Afterwards, a 1:100 dilution in fresh LB with 1% glucose was incubated at 37 °C shaking. When the culture reached an OD_600_ of 0.5, overexpression was induced by adding 10 mM IPTG and incubated for 3 h shaking at 37 °C. Cultures were spun down at 8,000 rpm for 10 min at 4 °C. Overexpression was confirmed performing an sodium dodecyl sulfate–polyacrylamide gel electrophoresis (SDS–PAGE) gel electrophoresis and including a negative control. For protein purification, the frozen pellets were resuspended in 50 mM sodium phosphate 300 mM sodium chloride buffer with one tablet of cOmplete Mini EDTA-free Protease Inhibitor Cocktail (Sigma-Aldrich). Samples were sonicated (Digital Sonier 450, Branson) and then centrifuged at 4,200*×g* for 10 min at 4 °C. Supernatant was recovered and then used for protein purification through Talon Metal Affinity resin (Clontech), as previously described (15). The presence of the purified protein was confirmed by loading the resulting fractions on an SDS–PAGE gel.

### Electrophoretic mobility shift assays

DNA probes were obtained designing 100 bp oligonucleotides pairs (Table S4) containing the predicted FddR-binding site with the desired nucleotides changes when required. An adenine (A) residue was added to both oligonucleotides 3′ end for pGEM-T vector (Promega) cloning. Ligation was introduced into *E. coli* DH5α competent cells and white colonies were screened by PCR using the M13 universal primers and then, confirmed by sequencing (Macrogen). Once the construction was obtained, DNA probes were labeled by PCR amplification using the universal M13 primers labeled at their 5′ ends with digoxigenin followed by PCR product gel band purification (GFX PCR DNA and Gel Band Purification Kit, Cytiva).

For EMSAs, labeled DNA probes (7 nM) and protein (from 0 to 120 nM) were mixed in binding buffer (10 mM 4-(2-hydroxyethyl)-1-piperazineethanesulfonic acid [HEPES], 10 mM Tris-HCl [pH 8], 1 mM EDTA, 50 mM KCl, 5% glycerol, 1 μg of bulk carrier salmon sperm DNA, 0.5 mM 1,4-dithiothreitol and 0.1 mg of bovine serum albumin per milliliter) and incubated at 30 °C for 30 min. When required, unlabeled promoter fragments were used as unlabeled DNA competitors in a 10-fold excess. Samples were loaded onto a 6% nondenaturing Tris-Glycine-EDTA polyacrylamide gel and DNA–protein complexes separated at 110 V for 90 min. Gel was blotted to a Biodine B nylon membrane (Pall Corporation), and the samples were fixed to the membrane using an UV Stratalinker 2400 (Stratagene). The membrane was incubated at room temperature for 30 min in blocking solution (1% skimmed milk powder phosphate-buffered saline [PBS] 1×) and then, with 20 mL of PBS 1× with 4 μL of Antidigoxigenin-AP Fab Fragment (Sigma-Aldrich) for 30 min. Afterward, the membrane was washed and developed in the dark with 5-Bromo-4-chloro-3-indolyl phosphate and 4-Nitro Blue Tetrazolium chloride in alkaline buffer (50 mM MgCl_2_, 6.6 mM Tris-HCl, 100 mM Trizma Base, 10 mM NaCl, pH 9.5).

### Motif discovery and regulon analysis

FSC454_RS03120 homologs were compiled from all *Francisella* spp. (*n* = 118) complete assemblies publicly available at GenBank (Table S5) through reciprocal BLASTP (42) using the FddR protein sequence (WP_066046500) as the query, a conservative *e*-value of <1*e*–20 and query coverage of >75%.

The upstream regions (from −250 to +2 bp of the predicted translational start site) of identified FSC454_RS03120 homologs were downloaded from the respective complete genome sequences. Redundant upstream sequences were removed with USEARCH with a 90% similarity threshold and otherwise default parameters (43), and the resulting panel was used to perform motif discovery. Motifs were inferred with MEME (44) using a 12- to 26-bp motif size, the palindrome arrangement setting, the any number of repetitions site distribution model, and otherwise default parameters.

Comparative genomics analyses of the regulatory networks defined by the identified motifs were conducted using the CGB platform, using COG and Pfam for the functional annotation of orthologous groups (15, 45). The CGB configuration file is provided in JSON format as Data S1.

### Phylogenetic inference

Protein sequences of nonredundant putative FddR homologs (identity <80%) predicted in the comparative genomics analyses were combined with S24 sequences from our previous study (15). As this dataset was predominantly composed of Bacteroidetes sequences, these were subsampled at a 70% identity threshold to minimize overrepresentation. To broaden the phylogenetic context and include phage-associated homologs, additional protein sequences were retrieved by BLASTP searches using the FddR (FSC454_RS03120) protein as the query. Searches were conducted against the NCBI nonredundant protein database excluding Francisellaceae, against the NCBI RefSeq Viruses and IMG/VR v4 databases (46). All searches were performed using an *e*-value of <1*e*−05, and up to 20 top-scoring hits were selected from each database for further analysis. Finally, 10 S26 peptidase protein sequences were randomly chosen from the Pfam database (PF10502) and included as an outgroup (47).

We then performed a T-COFFEE multiple sequence alignment using the previously described panel, combining three CLUSTALW protein sequence alignments with variable (5, 10, 25) gap open penalties (48). A maximum likelihood tree was constructed with IQ-TREE 2 (49) using 1,000 bootstrap replicates and LG + G4 as the substitution model, as deduced from ModelFinder (50). The resulting phylogenetic tree was visualized and annotated with the iTOL online tool (51).

## Supplementary Material

pgag114_Supplementary_Data

## Data Availability

RNA-seq data used in the study will be available in a persistent repository upon publication (GSE308416). All other data used in this study are included in the manuscript and its Supplementary material.
