# Supplementary material for: A phage repressor involved in the DNA-damage response of Francisella
Source: PNAS Nexus. 2026 Apr 17;5(5):pgag114. doi: 10.1093/pnasnexus/pgag114 (PMC13148647; doi:10.1093/pnasnexus/pgag114)
Supplement: pgag114_Supplementary_Data [file pgag114_supplementary_data.zip › PNASNEXUS-PNASNEXUS-2025-01710RR-s01.pdf]

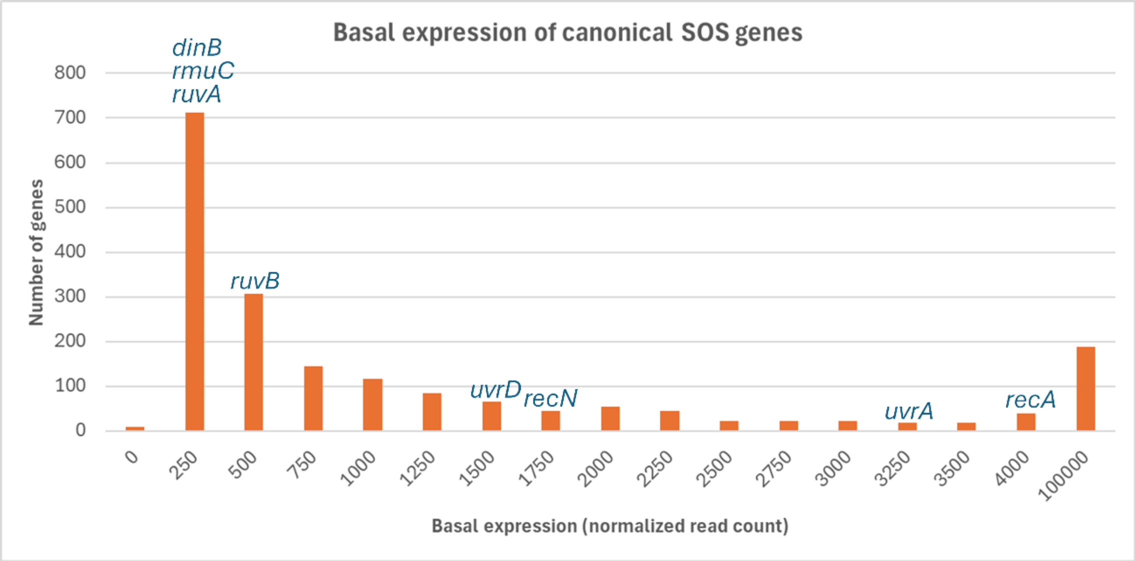

Supplementary Figure 1 – Histogram of basal expression levels in *F. hispaniensis*, highlighting the basal expression of canonical SOS genes (*dinB* – FSC454_RS04910, *recA* – FSC454_RS00525, *recN* – FSC454_RS01935, *rmuC* – FSC454_RS06825, *ruvA* – FSC454_RS04655, *ruvB* – FSC454_RS05755, *uvrA* – FSC454_RS06985 and *uvrD* – FSC454_RS08335). Reported normalized read counts are the average of three technical replicates.

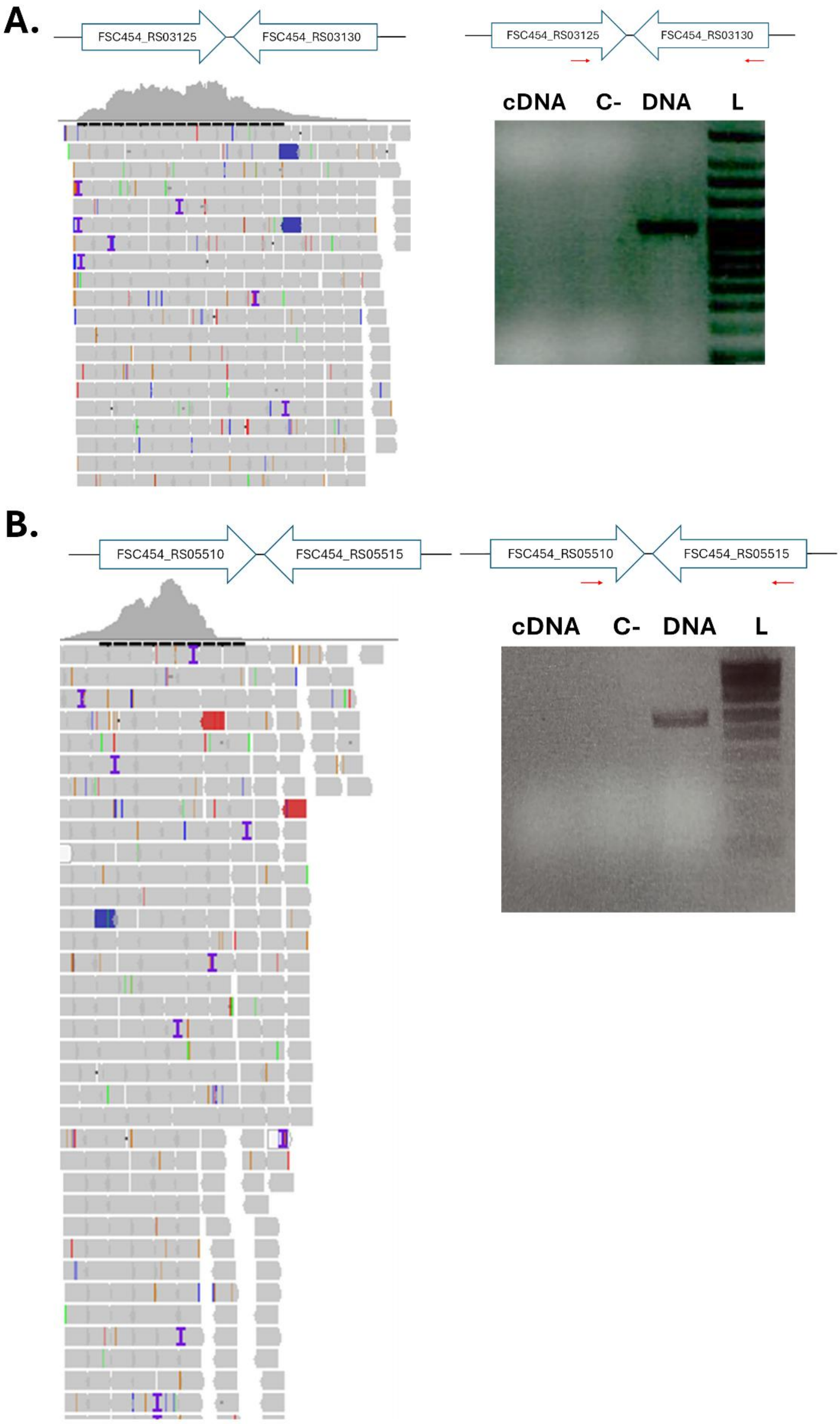

Supplementary Figure 2 – Analysis of transcriptional readthrough in gene pairs FSC454_RS03125-FSC454_RS03130 **(A)** and FSC454_RS05510-FSC454_RS05515 **(B)**, encoding a DMT family transporter and a deoxy-nucleoside triphosphate pyrophosphohydrolase, respectively. For each case, a section of the IGV-generated RNA-seq read distribution (left) and RT-PCR validation (right) are shown. The location of primers for RT-PCR is indicated by red arrows. The inability of the cDNA fragment to amplify, in contrast to the genomic DNA control, indicates that the distal region of readthrough genes is not co-transcribed

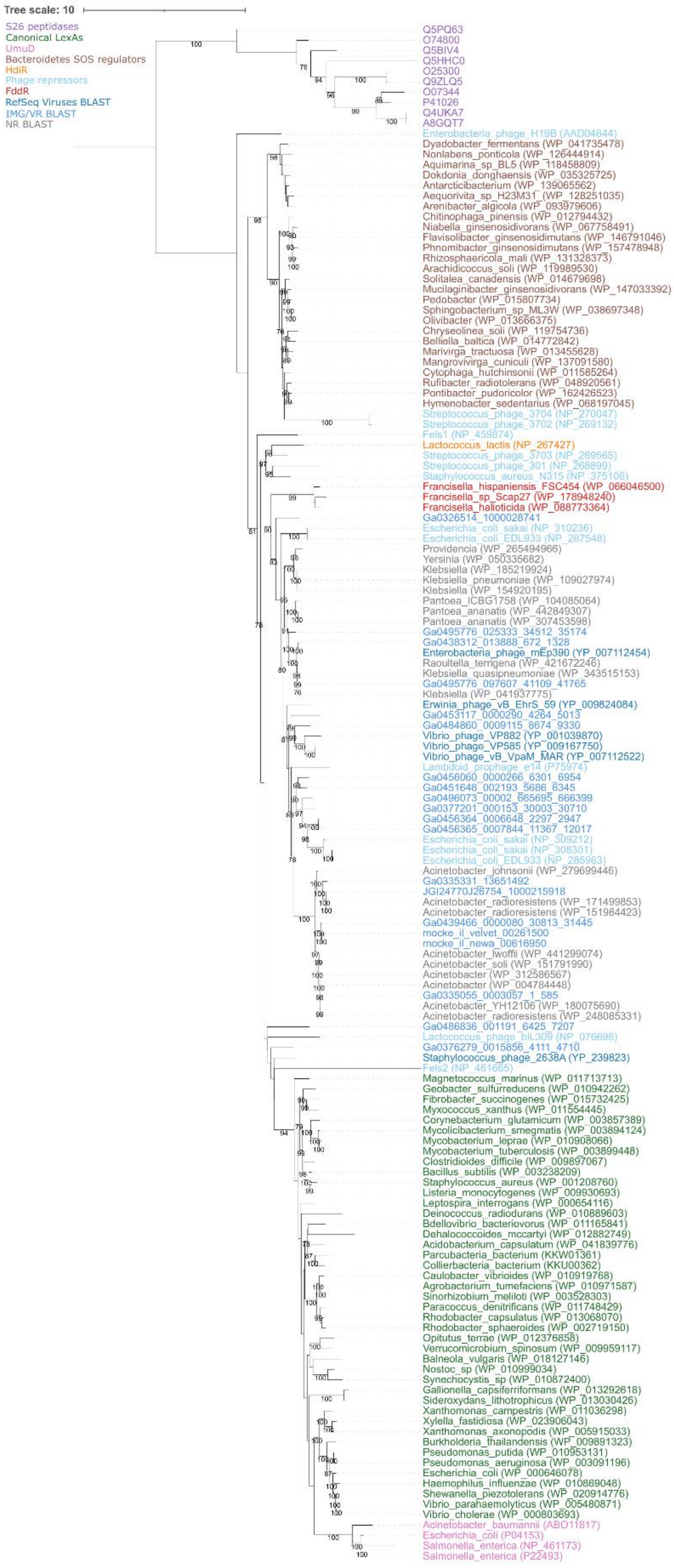

Supplementary Figure 3 - Rooted phylogenetic tree of S24 family peptidase family protein sequences. FddR homologs were combined with S24 family sequences from a previous study (Sánchez-Osuna et al., 2021). To broaden the phylogenetic context and incorporate phage-associated homologs, additional sequences were retrieved by BLASTP searches using FddR (FSC454_RS03120) as the query against the NCBI non-redundant database (excluding *Francisellaceae*), as well as the NCBI RefSeq Viruses and IMG/VR v4 databases. S26 peptidase representative sequences from the IPR015927 superfamily were included as the outgroup. Bootstrap support values are shown as percentages for nodes with support >75%.

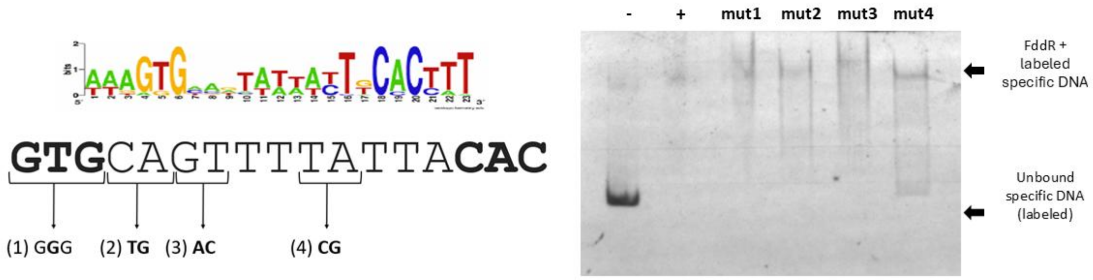

Supplementary Figure 4 – Electrophoretic mobility of DIG-labeled DNA fragments encompassing variants of the predicted FddR-binding site that did not show an effect on the electrophoretic mobility of the DNA-protein complex within the *fddR* promoter in the presence of purified FddR protein (12 mM). The FddR-binding motif inferred by MEME is shown as a sequence logo. Modifications to the FddR-binding site within the *fddR* promoter are indicated by arrows and numbers. The mobility of the wild-type DNA fragment in the absence (-) or presence (+) of the same amount of purified FddR protein is shown as a control.

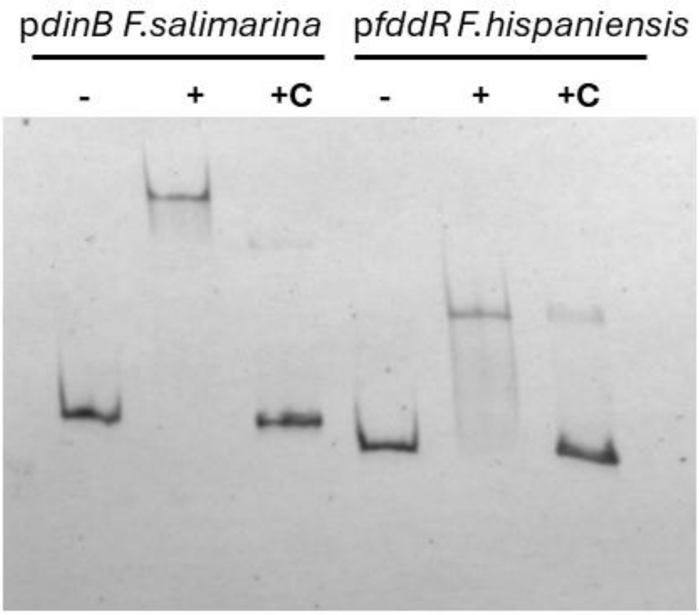

Supplementary Figure 5 – Reciprocal EMSAs using the purified *F. salimarina* FddR paralog and probes containing the upstream regions of the *F. salimarina dinB* and the *F. hispaniensis fddR* genes. The (-) symbol indicates absence of protein and the (+) symbol indicates presence of the purified S24 family peptidase. The (+C) symbol denotes the presence of unlabeled competitor DNA.

Supplementary Table 1 – RNA-seq values of the differentially expressed genes induced under ciprofloxacin (0.125 mg/L) treatment.

Locus tag	log2FoldChange	Accession	Begin	End	Orientation	Name	Symbol	Gene Type	Protein accession	Protein length
FSC454_RS03125	4,748092869	NZ_CP018093.1	627997	628428	plus	normocyte binding protein 2b		protein-coding	WP_066046502.1	143
FSC454_RS05510	4,644862882	NZ_CP018093.1	1121592	1121963	plus	hypothetical protein		protein-coding	WP_014548226.1	123
FSC454_RS05515	3,511107104	NZ_CP018093.1	1122167	1122571	minus	(deoxy)nucleoside triphosphate pyrophosphohydrolase		protein-coding	WP_066044912.1	134
FSC454_RS02345	3,353147119	NZ_CP018093.1	455010	455124	plus	5S ribosomal RNA	rrf	rRNA		
FSC454_RS03130	2,654141221	NZ_CP018093.1	628383	629249	minus	DMT family transporter		protein-coding	WP_066046504.1	288
FSC454_RS03120	2,405174551	NZ_CP018093.1	627176	627829	minus	S24 family peptidase		protein-coding	WP_066046500.1	217
FSC454_RS09600	1,92855869	NZ_CP018093.1	74751	74933	plus	hypothetical protein		protein-coding	WP_066045126.1	60
FSC454_RS00335	1,906597446	NZ_CP018093.1	71043	72587	minus	alkaline phosphatase family protein		protein-coding	WP_071794750.1	514
FSC454_RS04975	1,692920111	NZ_CP018093.1	1024230	1024829	plus	peroxiredoxin C		protein-coding	WP_014548376.1	199
FSC454_RS03775	1,678933676	NZ_CP018093.1	769580	769775	minus	6S RNA	ssrS	ncRNA		
FSC454_RS04935	1,636675105	NZ_CP018093.1	1016311	1018083	minus	ribonucleoside-diphosphate reductase subunit alpha		protein-coding	WP_066046287.1	590
FSC454_RS06550	1,630125062	NZ_CP018093.1	1332834	1333034	minus	30S ribosomal protein S21	rpsU	protein-coding	WP_014715333.1	66
FSC454_RS03405	1,581302347	NZ_CP018093.1	697788	697863	plus	tRNA-Ala		tRNA		
FSC454_RS08555	1,579608879		1740048	1741307	plus	citrate synthase		protein-coding	WP_066045251.1	419
FSC454_RS04980	1,395680064	NZ_CP018093.1	1024893	1026269	plus	hypothetical protein		protein-coding	WP_014548375.1	458
FSC454_RS03420	1,343812863	NZ_CP018093.1	701194	701490	plus	hypothetical protein		protein-coding	WP_066046449.1	98
FSC454_RS09605	1,33566979	NZ_CP018093.1	75085	75258	plus	hypothetical protein		protein-coding	WP_156470852.1	57
FSC454_RS03415	1,216407426	NZ_CP018093.1	700851	700965	plus	5S ribosomal RNA	rrf	rRNA		
FSC454_RS09115	1,210364311	NZ_CP018093.1	1865298	1865687	plus	uncharacterized gene		pseudogene		
FSC454_RS09455	1,208955807	NZ_CP018094.1	6008	6752	plus	uncharacterized gene		pseudogene		
FSC454_RS00345	1,183913102	NZ_CP018093.1	73568	74563	plus	6ystem6ic phosphate transporter		protein-coding	WP_014547410.1	331
FSC454_RS04930	1,171328264	NZ_CP018093.1	1016047	1016307	minus	glutaredoxin		protein-coding	WP_066046289.1	86
FSC454_RS09445	1,152020843	NZ_CP018094.1	4521	4853	minus	type II toxin-antitoxin 6ystem RelE/ParE family toxin		protein-coding	WP_156470919.1	110
FSC454_RS04220	1,127676138	NZ_CP018093.1	856481	857203	plus	SDR family oxidoreductase		protein-coding	WP_066047223.1	240
FSC454_RS03255	1,120495048	NZ_CP018093.1	657428	658474	minus	phosphate acyltransferase PlsX	plsX	protein-coding	WP_014548689.1	348
FSC454_RS01280	1,111138802	NZ_CP018093.1	246854	247231	minus	hypothetical protein		protein-coding	WP_066045865.1	125
FSC454_RS08905	1,105102178	NZ_CP018093.1	1818004	1818864	minus	aldose 1-epimerase family protein		protein-coding	WP_066046028.1	286

FSC454_RS02635	1,094856758	NZ_CP018093.1	529308	530474	plus	MFS transporter		protein-coding	WP_066044957.1	388
FSC454_RS00340	1,08159799	NZ_CP018093.1	72894	73550	plus	hypothetical protein		protein-coding	WP_014547409.1	218
FSC454_RS09035	1,066901987	NZ_CP018093.1	1850005	1850119	minus	5S ribosomal RNA	rrf	rRNA		
FSC454_RS10130	1,057787234	NZ_CP018093.1	1629832	1629963	minus	hypothetical protein		protein-coding	WP_003029898.1	43
FSC454_RS03270	1,053504097	NZ_CP018093.1	659437	661428	plus	transketolase	tkt	protein-coding	WP_066047502.1	663
FSC454_RS08055	1,052133671	NZ_CP018093.1	1630170	1630266	minus	signal recognition particle sRNA small type	ffs	other		
FSC454_RS03770	1,050360085	NZ_CP018093.1	769007	769555	minus	5-formyltetrahydrofolate cyclo-ligase		protein-coding	WP_066044657.1	182
FSC454_RS06670	1,034797447	NZ_CP018093.1	1355212	1355961	minus	hypothetical protein		protein-coding	WP_066046743.1	249
FSC454_RS00255	1,016468396	NZ_CP018093.1	57213	57461	minus	membrane protein insertion efficiency factor YidD	yidD	protein-coding	WP_197456246.1	82
FSC454_RS08305	1,005139161	NZ_CP018093.1	1679560	1680762	plus	MFS transporter		protein-coding	WP_066045311.1	400
FSC454_RS06455	-1,002157147	NZ_CP018093.1	1316929	1317165	minus	hypothetical protein		protein-coding	WP_066046217.1	78
FSC454_RS05020	-1,020008286	NZ_CP018093.1	1032423	1033919	plus	aldehyde dehydrogenase family protein		protein-coding	WP_066046259.1	498
FSC454_RS02560	-1,021105637	NZ_CP018093.1	511694	512476	minus	biotin--[acetyl-CoA-carboxylase] ligase		protein-coding	WP_066044879.1	260
FSC454_RS04655	-1,05004745	NZ_CP018093.1	956231	956881	plus	Holliday junction branch migration protein RuvA		protein-coding	WP_066045456.1	216
FSC454_RS06270	-1,055697895	NZ_CP018093.1	1279269	1279727	minus	ribonuclease HI	rnhA	protein-coding	WP_066046124.1	152
FSC454_RS00150	-1,080039398	NZ_CP018093.1	33672	34304	plus	orotidine-5'-phosphate decarboxylase	pyrF	protein-coding	WP_197456244.1	210
FSC454_RS09345	-1,10590748	NZ_CP018093.1	1908904	1909218	plus	hypothetical protein		protein-coding	WP_066045481.1	104
FSC454_RS04270	-1,109169595	NZ_CP018093.1	867954	868709	plus	HesA/MoeB/ThiF family protein		protein-coding	WP_014548508.1	251
FSC454_RS06175	-1,133797748	NZ_CP018093.1	1259434	1259859	plus	VirK family protein		protein-coding	WP_066047386.1	141
FSC454_RS04775	-1,153659495	NZ_CP018093.1	984841	985566	plus	CRISPR-associated endonuclease Cas1	cas1	protein-coding	WP_071794799.1	241
FSC454_RS09980	-1,156441383	NZ_CP018093.1	1010781	1010912	minus	uncharacterized gene		pseudogene		
FSC454_RS09420	-1,160312408	NZ_CP018093.1	1920622	1922118	minus	Rne/Rng family ribonuclease		protein-coding	WP_014549127.1	498
FSC454_RS05590	-1,163987171	NZ_CP018093.1	1138377	1139594	minus	GTP-binding protein		protein-coding	WP_066047445.1	405
FSC454_RS07450	-1,165902155	NZ_CP018093.1	1497909	1499201	minus	oligosaccharide repeat unit polymerase		protein-coding	WP_066046941.1	430
FSC454_RS06435	-1,182151698	NZ_CP018093.1	1313682	1314020	minus	hypothetical protein		protein-coding	WP_066046211.1	112
FSC454_RS08320	-1,196903634	NZ_CP018093.1	1682713	1683573	minus	ABC transporter permease		protein-coding	WP_014548935.1	286
FSC454_RS05435	-1,198139541	NZ_CP018093.1	1107110	1107886	minus	LysO family transporter		protein-coding	WP_071794802.1	258
FSC454_RS07105	-1,204249736	NZ_CP018093.1	1435629	1437134	minus	DUF3987 domain-containing protein		protein-coding	WP_066046233.1	501

FSC454_RS08460	-1,219904269	NZ_CP018093.1	1719857	1720840	plus	FAD-dependent oxidoreductase		protein-coding	WP_071794827.1	327
FSC454_RS00075	-1,223821535	NZ_CP018093.1	14141	17409	minus	uncharacterized gene	carB	pseudogene		
FSC454_RS06260	-1,223963945	NZ_CP018093.1	1277478	1278163	minus	uncharacterized gene		pseudogene		
FSC454_RS00155	-1,24253178	NZ_CP018093.1	34306	35048	plus	uncharacterized gene		pseudogene		
FSC454_RS01765	-1,24442106	NZ_CP018093.1	338873	339631	plus	sulfite exporter TauE/SafE family protein		protein-coding	WP_066045684.1	252
FSC454_RS04585	-1,249874555	NZ_CP018093.1	941668	945096	minus	transcription-repair coupling factor	mfd	protein-coding	WP_066046375.1	1142
FSC454_RS08455	-1,26082159	NZ_CP018093.1	1719414	1719860	plus	DUF3429 domain-containing protein		protein-coding	WP_044247504.1	148
FSC454_RS04250	-1,261262443	NZ_CP018093.1	864446	865498	plus	FAD-dependent oxidoreductase		protein-coding	WP_071794785.1	350
FSC454_RS08760	-1,26794765	NZ_CP018093.1	1785770	1787779	minus	NADH-quinone oxidoreductase subunit L	nuoL	protein-coding	WP_066046077.1	669
FSC454_RS09820	-1,281941584	NZ_CP018093.1	1742672	1742860	minus	hypothetical protein		protein-coding	WP_167359450.1	62
FSC454_RS04895	-1,282766766	NZ_CP018093.1	1009002	1009967	plus	transaldolase	tal	protein-coding	WP_066046295.1	321
FSC454_RS06265	-1,288057121	NZ_CP018093.1	1278230	1279267	minus	asparaginase	ansA	protein-coding	WP_066046121.1	345
FSC454_RS00070	-1,301384239	NZ_CP018093.1	13101	14024	minus	aspartate carbamoyltransferase	pyrB	protein-coding	WP_066045213.1	307
FSC454_RS05535	-1,311252677	NZ_CP018093.1	1124421	1126304	plus	DUF3857 domain-containing transglutaminase family protein		protein-coding	WP_071794804.1	627
FSC454_RS00720	-1,343042986	NZ_CP018093.1	145717	146607	plus	acyltransferase		protein-coding	WP_066045036.1	296
FSC454_RS08755	-1,377409094	NZ_CP018093.1	1784155	1785744	minus	NADH-quinone oxidoreductase subunit M		protein-coding	WP_066046080.1	529
FSC454_RS07850	-1,388405769	NZ_CP018093.1	1587850	1588734	minus	N-acetylmuramic acid 6-phosphate etherase	murQ	protein-coding	WP_066046826.1	294
FSC454_RS07440	-1,39013501	NZ_CP018093.1	1495750	1496862	minus	glycosyltransferase		protein-coding	WP_071794845.1	370
FSC454_RS05620	-1,39391648	NZ_CP018093.1	1145088	1145855	minus	nicotianamine synthase family protein		protein-coding	WP_167359445.1	255
FSC454_RS04120	-1,396398863	NZ_CP018093.1	835679	837793	plus	bifunctional (p)ppGpp synthetase/guanosine-3',5'-bis(diphosphate) 3'-pyrophosphohydrolase		protein-coding	WP_014548535.1	704
FSC454_RS07445	-1,411138257	NZ_CP018093.1	1496855	1497943	minus	glycosyltransferase		protein-coding	WP_066046939.1	362
FSC454_RS01755	-1,448787665	NZ_CP018093.1	337250	338179	plus	bile acid:sodium symporter family protein		protein-coding	WP_080555306.1	309
FSC454_RS05625	-1,469170089	NZ_CP018093.1	1146073	1147188	plus	MFS transporter		protein-coding	WP_231865178.1	371
FSC454_RS07430	-1,484188844	NZ_CP018093.1	1492607	1493851	minus	O-antigen translocase		protein-coding	WP_066046775.1	414
FSC454_RS04900	-1,592927916	NZ_CP018093.1	1009988	1010566	plus	hypothetical protein		protein-coding	WP_066046294.1	192

FSC454_RS07435	-1,60238894	NZ_CP018093.1	1493856	1495748	minus	asparagine synthase (glutamine-hydrolyzing)	asnB	protein-coding	WP_066046777.1	630
FSC454_RS00855	-1,606174155	NZ_CP018093.1	174865	176088	minus	MFS transporter		protein-coding	WP_066046330.1	407
FSC454_RS00015	-1,609874196	NZ_CP018093.1	2659	3918	minus	MFS transporter		protein-coding	WP_066044905.1	419
FSC454_RS10125	-1,614374442	NZ_CP018093.1	1556163	1556427	minus	uncharacterized gene		pseudogene		
FSC454_RS04180	-1,646382091	NZ_CP018093.1	846869	848932	plus	M13 family metallopeptidase		protein-coding	WP_066044761.1	687
FSC454_RS10025	-1,687642727	NZ_CP018093.1	1556586	1556726	minus	hypothetical protein		protein-coding	WP_244148256.1	46
FSC454_RS08750	-1,709377461	NZ_CP018093.1	1782688	1784139	minus	NADH-quinone oxidoreductase subunit N		protein-coding	WP_066046085.1	483
FSC454_RS03400	-1,745625171	NZ_CP018093.1	697697	697773	plus	tRNA-Ile		tRNA		

Supplementary Table 2 – List of FddR sequences identified via reciprocal BLASTP. Nucleotide identifiers, BLASTP e-values and query coverage are also provided.

**Organism_name**	**Nucleotide_Ids**	**FddR_Hit_evalue_coverage**
Francisella_adeliensis	NZ_CP043425	lcl|NZ_CP043425.1_prot_WP_112869352.1_299(e=4.61e-77,cov=96.0)
Francisella_adeliensis	NZ_CP043424	lcl|NZ_CP043424.1_prot_WP_112869352.1_299(e=4.61e-77,cov=96.0)
Francisella_adeliensis	NZ_CP021781	lcl|NZ_CP021781.1_prot_WP_112869352.1_299(e=4.61e-77,cov=96.0)
Francisella_cf_novicida_Fx1	NC_017450	lcl|NC_017450.1_prot_WP_014550072.1_1373(e=3.19e-144,cov=100.0)
Francisella_frigiditurris	NZ_CP009654<br>NZ_CP009655	
Francisella_halioticida	NZ_CP022132	lcl|NZ_CP022132.1_prot_WP_088773364.1_2362(e=4.11e-93,cov=98.0)
Francisella_halioticida	NZ_AP023082<br>NZ_AP023083<br>NZ_AP023084	lcl|NZ_AP023082.1_prot_WP_088773364.1_1911(e=4.31e-93,cov=98.0)
Francisella_hispaniensis	NC_017449	lcl|NC_017449.1_prot_WP_014548714.1_1418(e=2.35e-159,cov=100.0)
Francisella_hispaniensis_FSC454	NZ_CP018093<br>NZ_CP018094	lcl|NZ_CP018093.1_prot_WP_066046500.1_613(e=3.09e-163,cov=100.0)
Francisella_marina	NZ_CP043550<br>NZ_CP043551	lcl|NZ_CP043550.1_prot_WP_149368584.1_1102(e=1.12e-148,cov=100.0)
Francisella_marina	NZ_CP043552<br>NZ_CP043553	lcl|NZ_CP043552.1_prot_WP_149368584.1_315(e=1.13e-148,cov=100.0)
Francisella_noatunensis_subsp_noatunensis_FSC774	NZ_CP053850	lcl|NZ_CP053850.1_prot_WP_159184691.1_464(e=2.10e-152,cov=100.0)
Francisella_opportunistica	NZ_CP022376	lcl|NZ_CP022376.1_prot_WP_071629892.1_1695(e=1.25e-88,cov=98.0)
Francisella_opportunistica	NZ_CP022375	lcl|NZ_CP022375.1_prot_WP_071629892.1_1690(e=1.24e-88,cov=98.0)
Francisella_opportunistica	NZ_CP022377	lcl|NZ_CP022377.1_prot_WP_071629892.1_1704(e=1.25e-88,cov=98.0)
Francisella_orientalis	NZ_CP022947	lcl|NZ_CP022947.1_prot_WP_014714895.1_1443(e=2.53e-150,cov=100.0)
Francisella_orientalis	NZ_CP011923	lcl|NZ_CP011923.2_prot_WP_014714895.1_1459(e=2.54e-150,cov=100.0)
Francisella_orientalis	NZ_CP022948	lcl|NZ_CP022948.1_prot_WP_014714895.1_1458(e=2.54e-150,cov=100.0)
Francisella_orientalis	NZ_CP022945	lcl|NZ_CP022945.1_prot_WP_014714895.1_1457(e=2.54e-150,cov=100.0)
Francisella_orientalis	NZ_CP018051	lcl|NZ_CP018051.1_prot_WP_014714895.1_1457(e=2.53e-150,cov=100.0)
Francisella_orientalis	NZ_CP022942	lcl|NZ_CP022942.1_prot_WP_014714895.1_1459(e=2.53e-150,cov=100.0)
Francisella_orientalis	NZ_CP022946	lcl|NZ_CP022946.1_prot_WP_014714895.1_1455(e=2.53e-150,cov=100.0)
Francisella_orientalis	NZ_CP022950	lcl|NZ_CP022950.1_prot_WP_014714895.1_1458(e=2.54e-150,cov=100.0)
Francisella_orientalis	NZ_CP022939	lcl|NZ_CP022939.1_prot_WP_014714895.1_1448(e=2.53e-150,cov=100.0)
Francisella_orientalis	NZ_CP022941	lcl|NZ_CP022941.1_prot_WP_014714895.1_1462(e=2.53e-150,cov=100.0)
Francisella_orientalis	NZ_CP022944	lcl|NZ_CP022944.1_prot_WP_014714895.1_1459(e=2.54e-150,cov=100.0)
Francisella_orientalis	NZ_CP022938	lcl|NZ_CP022938.1_prot_WP_014714895.1_1462(e=2.54e-150,cov=100.0)
Francisella_orientalis	NZ_CP022940	lcl|NZ_CP022940.1_prot_WP_014714895.1_1460(e=2.54e-150,cov=100.0)
Francisella_orientalis	NZ_CP022949	lcl|NZ_CP022949.1_prot_WP_014714895.1_1459(e=2.54e-150,cov=100.0)
Francisella_orientalis	NZ_CP022943	lcl|NZ_CP022943.1_prot_WP_014714895.1_1458(e=2.54e-150,cov=100.0)
Francisella_orientalis	NZ_CP022953	lcl|NZ_CP022953.1_prot_WP_014714895.1_1462(e=2.54e-150,cov=100.0)
Francisella_orientalis	NZ_CP022952	lcl|NZ_CP022952.1_prot_WP_014714895.1_1465(e=2.54e-150,cov=100.0)
Francisella_orientalis	NZ_CP012153	lcl|NZ_CP012153.2_prot_WP_014714895.1_1461(e=2.54e-150,cov=100.0)

Francisella_orientalis	NZ_CP022951	lcl|NZ_CP022951.1_prot_WP_014714895.1_1462(e=2.54e-150,cov=100.0)
Francisella_orientalis_FNO12	NZ_CP011921	lcl|NZ_CP011921.2_prot_WP_014714895.1_1464(e=2.54e-150,cov=100.0)
Francisella_orientalis_FNO24	NZ_CP011922	lcl|NZ_CP011922.2_prot_WP_014714895.1_1462(e=2.54e-150,cov=100.0)
Francisella_orientalis_LADL_07-285A	NC_023029	lcl|NC_023029.1_prot_WP_014714895.1_1534(e=2.53e-150,cov=100.0)
Francisella_orientalis_str_Toba_04	NC_017909	lcl|NC_017909.1_prot_WP_014714895.1_942(e=2.53e-150,cov=100.0)
Francisella_persica_ATCC_VR-331	NZ_CP012505	lcl|NZ_CP012505.1_prot_ACH24_RS01090_234(e=3.00e-37,cov=76.0)
Francisella_persica_ATCC_VR-331	NZ_CP013022	lcl|NZ_CP013022.1_prot_FSC845_RS03950_831(e=3.04e-37,cov=76.0)
Francisella_philomiragia	NZ_CP009442<br>NZ_CP009443	lcl|NZ_CP009442.1_prot_WP_035736540.1_832(e=2.56e-154,cov=100.0)
Francisella_philomiragia	NZ_CP063138<br>NZ_CP063139	lcl|NZ_CP063139.1_prot_WP_211491149.1_14(e=1.14e-93,cov=100.0);lcl|NZ_CP063138.1_prot_WP_211490207.1_1292(e=3.24e-153,cov=100.0)
Francisella_philomiragia	NZ_CP009343<br>NZ_CP009342	lcl|NZ_CP009343.1_prot_WP_012280685.1_1525(e=2.84e-153,cov=100.0)
Francisella_philomiragia	NZ_CP009436<br>NZ_CP009437	lcl|NZ_CP009436.1_prot_WP_012280685.1_1942(e=2.85e-153,cov=100.0)
Francisella_philomiragia	NZ_CP009444<br>NZ_CP009446<br>NZ_CP009445	lcl|NZ_CP009444.1_prot_WP_042517802.1_950(e=1.24e-152,cov=100.0)
Francisella_philomiragia	NZ_CP009440<br>NZ_CP009441	lcl|NZ_CP009440.1_prot_WP_044526629.1_1523(e=2.49e-153,cov=100.0)
Francisella_philomiragia_subsp_philomiragia_ATCC_25015	NZ_CP010019	lcl|NZ_CP010019.1_prot_WP_004287343.1_658(e=8.32e-154,cov=100.0)
Francisella_salimarina	NZ_CP076680	lcl|NZ_CP076680.1_prot_WP_216691924.1_1378(e=6.34e-150,cov=100.0);lcl|NZ_CP076680.1_prot_WP_216692706.1_801(e=2.43e-93,cov=100.0)
Francisella_salina	NC_015696	lcl|NC_015696.1_prot_WP_013922720.1_938(e=4.13e-150,cov=100.0)
Francisella_sp_FSC1006	NZ_CP009574	
Francisella_sp_LA112445	NZ_CP041030	lcl|NZ_CP041030.1_prot_WP_192578796.1_1997(e=3.00e-91,cov=100.0)
Francisella_sp_MA067296	NZ_CP016930<br>NZ_CP016929	lcl|NZ_CP016930.1_prot_WP_071629892.1_1695(e=1.24e-88,cov=98.0)
Francisella_sp_Scap27	NZ_CP041326	lcl|NZ_CP041326.1_prot_WP_178948240.1_893(e=7.64e-77,cov=96.0)
Francisella_tularensis	CP073120	
Francisella_tularensis	CP063128	lcl|CP063128.1_prot_QOR27833.1_1347(e=3.04e-142,cov=100.0)
Francisella_tularensis	CP025778	lcl|CP025778.1_prot_AUP75119.1_654(e=3.22e-142,cov=100.0)
Francisella_tularensis	NZ_CP034467	lcl|NZ_CP034467.1_prot_WP_010032171.1_434(e=3.05e-142,cov=100.0)
Francisella_tularensis	NZ_CP034466	lcl|NZ_CP034466.1_prot_WP_010032171.1_503(e=3.05e-142,cov=100.0)
Francisella_tularensis	NZ_CP034468	lcl|NZ_CP034468.1_prot_WP_010032171.1_999(e=3.05e-142,cov=100.0)
Francisella_tularensis	NZ_CP066295	lcl|NZ_CP066295.1_prot_WP_010032171.1_657(e=3.05e-142,cov=100.0)
Francisella_tularensis	NZ_CP089548	lcl|NZ_CP089548.1_prot_WP_010032171.1_659(e=3.05e-142,cov=100.0)
Francisella_tularensis	NZ_CP073124	lcl|NZ_CP073124.1_prot_WP_003024039.1_1460(e=3.21e-143,cov=100.0)
Francisella_tularensis	NZ_CP089550	lcl|NZ_CP089550.1_prot_WP_003024039.1_1462(e=3.16e-143,cov=100.0)
Francisella_tularensis	NZ_CP073127	lcl|NZ_CP073127.1_prot_WP_014551210.1_1464(e=3.76e-143,cov=100.0)
Francisella_tularensis	NZ_CP073122	lcl|NZ_CP073122.1_prot_WP_014551210.1_1459(e=3.82e-143,cov=100.0)
Francisella_tularensis	NZ_CP073123	lcl|NZ_CP073123.1_prot_WP_014551210.1_1455(e=3.82e-143,cov=100.0)
Francisella_tularensis	NZ_CP073126	lcl|NZ_CP073126.1_prot_WP_014551210.1_1464(e=3.82e-143,cov=100.0)

Francisella_tularensis	NZ_CP073128	lcl|NZ_CP073128.1_prot_WP_014551210.1_1457(e=3.82e-143,cov=100.0)
Francisella_tularensis	NZ_CP073129	lcl|NZ_CP073129.1_prot_WP_014551210.1_1464(e=3.82e-143,cov=100.0)
Francisella_tularensis	NZ_CP073121	lcl|NZ_CP073121.1_prot_WP_014551210.1_1470(e=3.83e-143,cov=100.0)
Francisella_tularensis_subsp_holarctica	NZ_AP023460	lcl|NZ_AP023460.1_prot_WP_010032171.1_1456(e=3.07e-142,cov=100.0)
Francisella_tularensis_subsp_holarctica	NZ_AP023459	lcl|NZ_AP023459.1_prot_WP_010032171.1_248(e=3.07e-142,cov=100.0)
Francisella_tularensis_subsp_holarctica	NZ_CP044003	lcl|NZ_CP044003.1_prot_WP_010032171.1_1368(e=3.05e-142,cov=100.0)
Francisella_tularensis_subsp_holarctica	NZ_CP010289	lcl|NZ_CP010289.1_prot_WP_010032171.1_646(e=3.05e-142,cov=100.0)
Francisella_tularensis_subsp_holarctica	NZ_CP010288	lcl|NZ_CP010288.1_prot_WP_010032171.1_880(e=3.05e-142,cov=100.0)
Francisella_tularensis_subsp_holarctica	NZ_CP089549	lcl|NZ_CP089549.1_prot_WP_010032171.1_1365(e=3.01e-142,cov=100.0)
Francisella_tularensis_subsp_holarctica	NZ_CP009693	lcl|NZ_CP009693.1_prot_WP_010032171.1_15(e=3.05e-142,cov=100.0)
Francisella_tularensis_subsp_holarctica	NZ_CP044002	lcl|NZ_CP044002.1_prot_WP_010032171.1_1378(e=3.05e-142,cov=100.0)
Francisella_tularensis_subsp_holarctica	NZ_CP044004	lcl|NZ_CP044004.1_prot_WP_010032171.1_1376(e=3.05e-142,cov=100.0)
Francisella_tularensis_subsp_holarctica	NZ_CP058275	lcl|NZ_CP058275.1_prot_WP_010032171.1_660(e=3.05e-142,cov=100.0)
Francisella_tularensis_subsp_holarctica	NZ_CP044005	lcl|NZ_CP044005.1_prot_WP_010032171.1_1377(e=3.05e-142,cov=100.0)
Francisella_tularensis_subsp_holarctica	NZ_CP073125	lcl|NZ_CP073125.1_prot_WP_010032171.1_1459(e=3.05e-142,cov=100.0)
Francisella_tularensis_subsp_holarctica	NZ_CP058301	lcl|NZ_CP058301.1_prot_WP_010032171.1_657(e=3.05e-142,cov=100.0)
Francisella_tularensis_subsp_holarctica	NZ_CP098826	lcl|NZ_CP098826.1_prot_WP_010032171.1_658(e=3.06e-142,cov=100.0)
Francisella_tularensis_subsp_holarctica	NZ_CP048229	lcl|NZ_CP048229.1_prot_WP_010032171.1_660(e=3.05e-142,cov=100.0)
Francisella_tularensis_subsp_holarctica	NZ_CP058274	lcl|NZ_CP058274.1_prot_WP_010032171.1_660(e=3.05e-142,cov=100.0)
Francisella_tularensis_subsp_holarctica_F92	NC_019537	lcl|NC_019537.1_prot_WP_010032171.1_654(e=3.04e-142,cov=100.0)
Francisella_tularensis_subsp_holarctica_FSC200	NC_019551	lcl|NC_019551.1_prot_WP_010032171.1_659(e=3.05e-142,cov=100.0)
Francisella_tularensis_subsp_holarctica_FTNF002-00	NC_009749	lcl|NC_009749.1_prot_WP_010032171.1_662(e=3.05e-142,cov=100.0)
Francisella_tularensis_subsp_holarctica_LVS	NC_007880	lcl|NC_007880.1_prot_WP_010032171.1_659(e=3.06e-142,cov=100.0)
Francisella_tularensis_subsp_holarctica_LVS	NZ_CP009694	lcl|NZ_CP009694.1_prot_WP_010032171.1_1378(e=3.05e-142,cov=100.0)
Francisella_tularensis_subsp_holarctica_OSU18	NC_017463	lcl|NC_017463.1_prot_WP_010032171.1_659(e=3.06e-142,cov=100.0)
Francisella_tularensis_subsp_holarctica_OSU18	NC_008369	lcl|NC_008369.1_prot_WP_010032171.1_659(e=3.06e-142,cov=100.0)
Francisella_tularensis_subsp_holarctica_PHIT-FT049	NZ_CP007148	lcl|NZ_CP007148.1_prot_WP_010032171.1_654(e=3.02e-142,cov=100.0)
Francisella_tularensis_subsp_mediasiatica_FSC147	CP000915	lcl|CP000915.1_prot_ACD30610.1_491(e=3.29e-143,cov=100.0)
Francisella_tularensis_subsp_novicida	NZ_CP009653	lcl|NZ_CP009653.1_prot_WP_032729592.1_1379(e=3.26e-144,cov=100.0)
Francisella_tularensis_subsp_novicida	NZ_CP009682 NZ_CP009683	lcl|NZ_CP009682.1_prot_WP_003040221.1_1360(e=1.12e-144,cov=100.0)
Francisella_tularensis_subsp_novicida	NZ_CP021490	lcl|NZ_CP021490.1_prot_WP_173677749.1_628(e=8.18e-142,cov=100.0)
Francisella_tularensis_subsp_novicida	NZ_CP010103 NZ_CP010104	lcl|NZ_CP010103.1_prot_WP_032729592.1_557(e=3.46e-144,cov=100.0)
Francisella_tularensis_subsp_novicida_D9876	NZ_CP009607	lcl|NZ_CP009607.1_prot_WP_003037214.1_1260(e=7.57e-145,cov=100.0)
Francisella_tularensis_subsp_novicida_F6168	NZ_CP009353 NZ_CP009352	lcl|NZ_CP009353.1_prot_WP_032729592.1_1102(e=3.28e-144,cov=100.0)
Francisella_tularensis_subsp_novicida_PA10-7858	NZ_CP016635	lcl|NZ_CP016635.1_prot_WP_003037214.1_1470(e=8.01e-145,cov=100.0);lcl|NZ_CP016635.1_prot_WP_071304469.1_1195(e=1.62e-55,cov=97.0)
Francisella_tularensis_subsp_novicida_U112	NC_008601	lcl|NC_008601.1_prot_WP_003040221.1_1376(e=1.11e-144,cov=100.0)
Francisella_tularensis_subsp_novicida_U112	NZ_CP009633	lcl|NZ_CP009633.1_prot_WP_003040221.1_616(e=1.11e-144,cov=100.0)

Francisella_tularensis_subsp_tularensis	NZ_CP010115	lcl|NZ_CP010115.1_prot_WP_003024039.1_811(e=3.17e-143,cov=100.0)
Francisella_tularensis_subsp_tularensis	NZ_CP012037	lcl|NZ_CP012037.1_prot_WP_003024039.1_1305(e=3.23e-143,cov=100.0)
Francisella_tularensis_subsp_tularensis	NZ_CP058276	lcl|NZ_CP058276.1_prot_WP_014551210.1_1457(e=3.82e-143,cov=100.0)
Francisella_tularensis_subsp_tularensis_FSC198	NC_008245	lcl|NC_008245.1_prot_WP_014551210.1_1455(e=3.82e-143,cov=100.0)
Francisella_tularensis_subsp_tularensis_MA00-2987	NZ_CP012372	lcl|NZ_CP012372.1_prot_WP_003024039.1_1458(e=3.21e-143,cov=100.0)
Francisella_tularensis_subsp_tularensis_NE061598	NC_017453	lcl|NC_017453.1_prot_WP_014551210.1_1405(e=3.82e-143,cov=100.0)
Francisella_tularensis_subsp_tularensis_SCHU_S4	NC_006570	lcl|NC_006570.2_prot_WP_014551210.1_1455(e=3.82e-143,cov=100.0)
Francisella_tularensis_subsp_tularensis_SCHU_S4	NZ_CP010290	lcl|NZ_CP010290.1_prot_WP_014551210.1_1264(e=3.82e-143,cov=100.0)
Francisella_tularensis_subsp_tularensis_str_SCHU_S4_substr_NR-28534	NZ_CP010446<br>NZ_CP010447	lcl|NZ_CP010446.2_prot_WP_014551210.1_1456(e=3.81e-143,cov=100.0)
Francisella_tularensis_subsp_tularensis_TI0902	NC_016937	lcl|NC_016937.2_prot_WP_003024039.1_1466(e=3.21e-143,cov=100.0)
Francisella_tularensis_subsp_tularensis_TIGB03	NC_016933	lcl|NC_016933.2_prot_WP_003024039.1_1555(e=3.34e-143,cov=100.0)
Francisella_tularensis_subsp_tularensis_WY-00W4114	NZ_CP009753	lcl|NZ_CP009753.1_prot_WP_003024039.1_450(e=3.23e-143,cov=100.0)
Francisella_tularensis_subsp_tularensis_WY96-3418	NC_009257	lcl|NC_009257.1_prot_WP_003024039.1_448(e=3.23e-143,cov=100.0)
Francisella_uliginis	NZ_CP016796	lcl|NZ_CP016796.1_prot_WP_072713479.1_2021(e=1.37e-93,cov=100.0)

Table S3 – Prediction of prophages on the nucleotide sequences encoding FddR homologs in *Francisella* and in Gammaproteobacteria.

Item	Species	Locus_tag	Function	protein_id	contig	PhiSpy_prophage
FddR	*Francisella halioticida*	CDV26_RS11440	LexA family protein	WP_088773364	NZ_CP022132.1	0
FddR	*Francisella sp.*	FLM55_RS04510	LexA family protein	WP_178948240	NZ_CP041326.1	0
FddR	*Francisella hispaniensis*	FSC454_RS03120	LexA family protein	WP_066046500	NZ_CP018093.1	0
NR_BLAST	*Klebsiella variicola*	AAH455_RS07700	LexA family protein	WP_041937775	NZ_CP153665.1	1
NR_BLAST	*Klebsiella michiganensis*	ABDO74_RS06495	LexA family protein	WP_154920195	NZ_JAYDVX010000002.1	1
NR_BLAST	*Klebsiella quasipneumoniae*	ABDR09_RS22130	LexA family protein	WP_343515153	NZ_JAYDGH010000014.1	0
NR_BLAST	*Acinetobacter radioresistens*	ACLIL3_RS03015	LexA family protein	WP_151984423	NZ_JBKJAK020000005.1	1
NR_BLAST	*Acinetobacter radioresistens*	ACNGTX_RS14865	LexA family protein	WP_248085331	NZ_JBLTHO010000026.1	0
NR_BLAST	*Raoultella terrigena*	ACO0U2_RS04900	LexA family protein	WP_421672246	NZ_JBLZOC010000001.1	1
NR_BLAST	*Acinetobacter lwoffii*	ACTL4S_RS07705	LexA family protein	WP_441299074	NZ_JBQGUZ010000009.1	0
NR_BLAST	*Pantoea ananatis*	ACTUSN_RS03580	LexA family protein	WP_442849307	NZ_JBQOUP010000002.1	1
NR_BLAST	*Pantoea sp.*	C1Y41_RS05885	LexA family protein	WP_104085064	NZ_POWL01000001.1	1
NR_BLAST	*Acinetobacter variabilis*	F969_RS12015	LexA family protein	WP_004784448	NZ_KB849404.1	1
NR_BLAST	*Klebsiella pneumoniae*	FOZ68_RS06810	LexA family protein	WP_109027974	NZ_CP041946.1	0
NR_BLAST	*Acinetobacter sp.*	FTU45_RS00205	LexA family protein	WP_180075690	NZ_VPAV01000001.1	1
NR_BLAST	*Acinetobacter radioresistens*	GB161_RS01525	LexA family protein	WP_171499853	NZ_BKZJ01000001.1	1
NR_BLAST	*Acinetobacter soli*	GBP85_RS16260	LexA family protein	WP_151791990	NZ_BKPF01000023.1	0
NR_BLAST	*Klebsiella michiganensis*	H5403_RS07665	LexA family protein	WP_185219924	NZ_CP060111.1	0
NR_BLAST	*Providencia sp.*	KA516_RS01680	LexA family protein	WP_265494966	NZ_JAGNYJ010000002.1	1
NR_BLAST	*Acinetobacter johnsonii*	N5I15_RS01200	S24 family peptidase	WP_279699446	NZ_JAOCHM010000002.1	1
NR_BLAST	*Pantoea ananatis*	QE443_RS13005	LexA family protein	WP_307453598	NZ_JAUTBM010000003.1	1
NR_BLAST	*Yersinia enterocolitica*	QJR53_RS07740	LexA family protein	WP_050335682	NZ_CP124238.1	0
NR_BLAST	*Acinetobacter sp.*	RTS85_RS11835	LexA family protein	WP_312586567	NZ_DALZPJ010000023.1	0

Table S4 – Oligonucleotides sequences used in this work.

**Oligonucleotide**	**Sequence**	**Use**
Fh *FSC454_RS03120* XhoI F	ATCG<u>CTCGAG</u>ATGTTTGACTAC	Gene cloning for protein overexpression
Fh *FSC454_RS03120* BamHI R	AG<u>GGATCC</u>TTAGAAGTGTCTG	Gene cloning for protein overexpression
tul4 F.h. qRT-PCR F	CTGCAACTATGCAAGCTACT	qRT-PCR
tul4 F.h. qRT-PCR R	GTATCATGGCACTTAGAACCT	qRT-PCR
FSC454_RS03120 F.h. qRT-PCR F	AAGCTTGGTAAAAGTGATCG	qRT-PCR
FSC454_RS03120 F.h. qRT-PCR R	AATTCACCAGCCTGAACATA	qRT-PCR
FSC454_RS00525 F.h. qRT-PCR F	GAGCAGAAATTGAAGGTGAC	qRT-PCR
FSC454_RS00525 F.h. qRT-PCR R	CATTACCACCAGTTGTAGTCTC	qRT-PCR
Fh prom wt F	ATAATACTTCCCTTTATGGCAAAAAGTAGTTAGTATTGAAGGTGTAATAAAACTGCACTTTTATAAGTTGAGGGAGTCAAACAAAATGATAAAAAAAAGA	EMSA probe
Fh prom wt R	CTTTTTTTTATCATTTTGTTTGACTCCCTCAACTTATAAAAGTGCAGTTTTATTACACCTTCAATACTAACTACTTTTTGCCATAAAGGGAAGTATTATA	EMSA probe
Fp *dinB* prom wt F	TATCAATATTAAAAGTGAAATAATACTTCACATTTTGAAGGTTATCATCTATATTTTTAATAAGTGAAATTATACTACACCTTATTAAGGCTAATATATA	EMSA probe
Fp *dinB* prom wt R	ATATATTAGCCTTAATAAGGTGTAGTATAATTTCACTTATTAAAAATATAGATGATAACCTTCAAAATGTGAAGTATTATTTCACTTTTAATATTGATAA	EMSA probe
Fs *dinB* prom wt F	TGTATCAATATTAAAAGTGAAATAATACTTCACATTTAAGGGTTTATCATCTATATTTTTAATAAGTGAAGTAAAACTACACTTTATTGAGGATGATATA	EMSA probe
Fs *dinB* prom wt R	ATATCATCCTCAATAAAGTGTAGTTTTACTTCACTTATTAAAAATATAGATGATAAACCCTTAAATGTGAAGTATTATTTCACTTTTAATATTGATACAA	EMSA probe
Fh prom mut 1 F	ATAATACTTCCCTTTATGGCAAAAAGTAGTTAGTATTGAAGGTGTAATAAAACTGTGTTTTTATAAGTTGAGGGAGTCAAACAAAATGATAAAAAAAAGA	Mutant EMSA probe
Fh prom mut 1 R	CTTTTTTTTATCATTTTGTTTGACTCCCTCAACTTATAAAAACACAGTTTTATTACACCTTCAATACTAACTACTTTTTGCCATAAAGGGAAGTATTATA	Mutant EMSA probe
Fh prom mut 2 F	ATAATACTTCCCTTTATGGCAAAAAGTAGTTAGTATTGAAGGTGTAATAAAACTGCCCTTTTATAAGTTGAGGGAGTCAAACAAAATGATAAAAAAAAGA	Mutant EMSA probe
Fh prom mut 2 R	CTTTTTTTTATCATTTTGTTTGACTCCCTCAACTTATAAAAGGGCAGTTTTATTACACCTTCAATACTAACTACTTTTTGCCATAAAGGGAAGTATTATA	Mutant EMSA probe
Fh prom mut 3 F	ATAATACTTCCCTTTATGGCAAAAAGTAGTTAGTATTGAAGGTGTAATAAAACCACACTTTTATAAGTTGAGGGAGTCAAACAAAATGATAAAAAAAAGA	Mutant EMSA probe
Fh prom mut 3 R	CTTTTTTTTATCATTTTGTTTGACTCCCTCAACTTATAAAAGTGTGGTTTTATTACACCTTCAATACTAACTACTTTTTGCCATAAAGGGAAGTATTATA	Mutant EMSA probe
Fh prom mut 4 F	ATAATACTTCCCTTTATGGCAAAAAGTAGTTAGTATTGAAGGTGTAATAAAGTTGCACTTTTATAAGTTGAGGGAGTCAAACAAAATGATAAAAAAAAGA	Mutant EMSA probe
Fh prom mut 4 R	CTTTTTTTTATCATTTTGTTTGACTCCCTCAACTTATAAAAGTGCAACTTTATTACACCTTCAATACTAACTACTTTTTGCCATAAAGGGAAGTATTATA	Mutant EMSA probe

Fh prom mut 5 F	TAATACTTCCCTTTATGGCAAAAAGTAGTTAGTATTGAAGGTGTAATAACCAACTGCACTTTTATAAGTTGAGGGAGTCAAACAAAATGATAAAAAAAAA	Mutant EMSA probe
Fh prom mut 5 R	TTTTTTTTATCATTTTGTTTGACTCCCTCAACTTATAAAAGTGCAGTTGGTTATTACACCTTCAATACTAACTACTTTTTGCCATAAAGGGAAGTATTAA	Mutant EMSA probe
Fh prom mut 6 F	AATACTTCCCTTTATGGCAAAAAGTAGTTAGTATTGAAGGTGTAATAACCCAACTGCACTTTTATAAGTTGAGGGAGTCAAACAAAATGATAAAAAAAAA	Mutant EMSA probe
Fh prom mut 6 R	TTTTTTTTATCATTTTGTTTGACTCCCTCAACTTATAAAAGTGCAGTTGGGTTATTACACCTTCAATACTAACTACTTTTTGCCATAAAGGGAAGTATTA	Mutant EMSA probe
Fh prom mut 7 F	ATAATACTTCCCTTTATGGCAAAAAGTAGTTAGTATTGAAGGTGTAACGAAACTGCACTTTTATAAGTTGAGGGAGTCAAACAAAATGATAAAAAAAAGA	Mutant EMSA probe
Fh prom mut 7 R	CTTTTTTTTATCATTTTGTTTGACTCCCTCAACTTATAAAAGTGCAGTTTCGTTACACCTTCAATACTAACTACTTTTTGCCATAAAGGGAAGTATTATA	Mutant EMSA probe
Fh prom mut 8 F	ATAATACTTCCCTTTATGGCAAAAAGTAGTTAGTATTGAAGCCCTAATAAAACTGCACTTTTATAAGTTGAGGGAGTCAAACAAAATGATAAAAAAAAGA	Mutant EMSA probe
Fh prom mut 8 R	CTTTTTTTTATCATTTTGTTTGACTCCCTCAACTTATAAAAGTGCAGTTTTATTAGGGCTTCAATACTAACTACTTTTTGCCATAAAGGGAAGTATTATA	Mutant EMSA probe
Fp *dinB* prom mut F	TATCAATATTAAAACCCAAATAATACTTTGTATTTTGAAGGTTATCATCTATATTTTTAATAACCCAAATTATACTATGTCTTATTAAGGCTAATATATA	Mutant EMSA probe
Fp *dinB* prom mut R	ATATATTAGCCTTAATAAGACATAGTATAATTTGGGTTATTAAAAATATAGATGATAACCTTCAAAATACAAAGTATTATTTGGGTTTTAATATTGATAA	Mutant EMSA probe
Fs *dinB* prom mut F	TGTATCAATATTAAAACCCAAATAATACTTTGTATTTAAGGGTTTATCATCTATATTTTTAATAACCCAAGTAAAACTATGTTTTATTGAGGATGATATA	Mutant EMSA probe
Fs *dinB* prom mut R	ATATCATCCTCAATAAAACATAGTTTTACTTGGGTTATTAAAAATATAGATGATAAACCCTTAAATACAAAGTATTATTTGGGTTTTAATATTGATACAA	Mutant EMSA probe
RT-PCR *FSC454_RS03125-FSC454_RS03130* F	GAGTTGCATAATTCAATTAAG	RT-PCR
RT-PCR *FSC454_RS03125-FSC454_RS03130* R	ATGAATAGAGATTTTTTCATAA	RT-PCR
RT-PCR *FSC454_RS05510-FSC454_RS05515* F	CTTAATGATGATACAACAG	RT-PCR
RT-PCR *FSC454_RS05510-FSC454_RS05515* R	ATGGCACATATTAATGCC	RT-PCR

Table S5 – List of complete NCBI Francisella genomes (n=118) analyzed in this study, including strain information, assembly statistics, and database access links.

Organism Name	Strain	BioSample	BioProject	Assembly	Level	Size(Mb)	GC%	Replicons	CDS	Release Date	GenBank FTP	RefSeq FTP
Francisella adeliensis	FSC1326	SAMN12641259	PRJNA562469	GCA_012224275.1	Complete	205.417	32.7	chromosome:NZ_CP043425.1/CP043425.1	1886	2020-04-09T00:00:00Z	ftp://ftp.ncbi.nlm.nih.gov/genomes/all/GCA/012/224/275/GCA_012224275.1_ASM1222427v1	ftp://ftp.ncbi.nlm.nih.gov/genomes/all/GCF/012/224/275/GCF_012224275.1_ASM1222427v1
Francisella adeliensis	FSC1325	SAMN12641260	PRJNA562468	GCA_012224465.1	Complete	205.423	32.7	chromosome:NZ_CP043424.1/CP043424.1	1888	2020-04-09T00:00:00Z	ftp://ftp.ncbi.nlm.nih.gov/genomes/all/GCA/012/224/465/GCA_012224465.1_ASM1222446v1	ftp://ftp.ncbi.nlm.nih.gov/genomes/all/GCF/012/224/465/GCF_012224465.1_ASM1222446v1
Francisella adeliensis	FDC440	SAMN07192706	PRJNA389235	GCA_003290445.1	Complete	205.409	32.7	chromosome:NZ_CP021781.1/CP021781.1	1888	2018-07-09T00:00:00Z		ftp://ftp.ncbi.nlm.nih.gov/genomes/all/GCF/003/290/445/GCF_003290445.1_ASM329044v1
Francisella cf. novicida Fx1	Fx1	SAMN02603513	PRJNA62751	GCA_000195535.1	Complete	191.362	32.5	chromosome:NC_017450.1/CP002557.1	1744	2011-04-08T00:00:00Z	ftp://ftp.ncbi.nlm.nih.gov/genomes/all/GCA/000/195/535/GCA_000195535.1_ASM19553v1	ftp://ftp.ncbi.nlm.nih.gov/genomes/all/GCF/000/195/535/GCF_000195535.1_ASM19553v1
Francisella frigiditurris	CA97-1460	SAMN03107515	PRJNA260085	GCA_001880225.1	Complete	186.161	312.017	chromosome:NZ_CP009654.1/CP009654.1; plasmid pFCD_1:NZ_CP009655.1/CP009655.1	1777	2016-11-17T00:00:00Z		ftp://ftp.ncbi.nlm.nih.gov/genomes/all/GCF/001/880/225/GCF_001880225.1_ASM188022v1
Francisella halioticida	DSM 23729	SAMN07207140	PRJNA389776	GCA_002211785.1	Complete	219.743	31.2	chromosome:NZ_CP022132.1/CP022132.1	2231	2017-06-30T00:00:00Z		ftp://ftp.ncbi.nlm.nih.gov/genomes/all/GCF/002/211/785/GCF_002211785.1_ASM221178v1

Francisella halioticida	UTH170823	SAMD00222553	PRJDB9717	GCA_018326105.1	Complete	229.856	311.879	chromosome:NZ_AP023082.1/AP023082.1; plasmid pFHSM1:NZ_AP023083.1/AP023083.1; plasmid pFHSM2:NZ_AP023084.1/AP023084.1	2369	2021-03-17T00:00:00Z	ftp://ftp.ncbi.nlm.nih.gov/genomes/all/GCA/018/326/105/GCA_018326105.1_ASM1832610v1	ftp://ftp.ncbi.nlm.nih.gov/genomes/all/GCF/018/326/105/GCF_018326105.1_ASM1832610v1
Francisella hispaniensis	3523	SAMN02603514	PRJNA62753	GCA_000195555.1	Complete	194.531	32.3	chromosome:NC_017449.1/CP002558.1	1809	2011-04-08T00:00:00Z	ftp://ftp.ncbi.nlm.nih.gov/genomes/all/GCA/000/195/555/GCA_000195555.1_ASM19555v1	ftp://ftp.ncbi.nlm.nih.gov/genomes/all/GCF/000/195/555/GCF_000195555.1_ASM19555v1
Francisella hispaniensis FSC454	CCUG 58020	SAMN00993432	PRJNA73391	GCA_001885235.1	Complete	193.864	321.479	chromosome:NZ_CP018093.1/CP018093.1; plasmid pFSC454:NZ_CP018094.1/CP018094.1	1744	2016-11-22T00:00:00Z		ftp://ftp.ncbi.nlm.nih.gov/genomes/all/GCF/001/885/235/GCF_001885235.1_ASM188523v1
Francisella marina	E103-15	SAMN12675350	PRJNA563510	GCA_008369765.1	Complete	20.453	328.928	chromosome:NZ_CP043550.1/CP043550.1; plasmid pE103_15_1:NZ_CP043551.1/CP043551.1	1949	2019-09-11T00:00:00Z		ftp://ftp.ncbi.nlm.nih.gov/genomes/all/GCF/008/369/765/GCF_008369765.1_ASM836976v1
Francisella marina	E95-16	SAMN12675351	PRJNA563512	GCA_008369785.1	Complete	206.228	328.928	chromosome:NZ_CP043552.1/CP043552.1; plasmid pE95_16_1:NZ_CP043553.1/CP043553.1	1959	2019-09-11T00:00:00Z		ftp://ftp.ncbi.nlm.nih.gov/genomes/all/GCF/008/369/785/GCF_008369785.1_ASM836978v1
Francisella noatunensis subsp. noatunensis FSC774	FSC774	SAMN00993444	PRJNA73457	GCA_014844275.1	Complete	187.055	32.5	chromosome:NZ_CP053850.1/CP053850.1	1873	2020-10-05T00:00:00Z	ftp://ftp.ncbi.nlm.nih.gov/genomes/all/GCA/014/844/275/GCA_014844275.1_ASM1484427v1	ftp://ftp.ncbi.nlm.nih.gov/genomes/all/GCF/014/844/275/GCF_014844275.1_ASM1484427v1
Francisella opportunistica	VA14-2155	SAMN07290515	PRJNA392307	GCA_003347115.1	Complete	18.313	32.5	chromosome:NZ_CP022376.1/CP022376.1	1675	2018-07-31T00:00:00Z		ftp://ftp.ncbi.nlm.nih.gov/genomes/all/GCF/003/347/11

												5/GCF_003347115.1_ASM334711v1
Francisella opportunistica	14-2155	SAMN07290516	PRJNA392307	GCA_003347095.1	Complete	182.497	32.5	chromosome:NZ_CP022375.1/CP022375.1	1678	2018-07-31T00:00:00Z		ftp://ftp.ncbi.nlm.nih.gov/genomes/all/GCF/003/347/095/GCF_003347095.1_ASM334709v1
Francisella opportunistica	VA14-2155	SAMN07290514	PRJNA392307	GCA_003347135.1	Complete	183.923	32.5	chromosome:NZ_CP022377.1/CP022377.1	1690	2018-07-31T00:00:00Z		ftp://ftp.ncbi.nlm.nih.gov/genomes/all/GCF/003/347/135/GCF_003347135.1_ASM334713v1
Francisella orientalis	FNO137	SAMN02666527	PRJNA240881	GCA_008330405.1	Complete	186.221	32.3	chromosome:NZ_CP022947.1/CP022947.1	1778	2019-09-09T00:00:00Z		ftp://ftp.ncbi.nlm.nih.gov/genomes/all/GCF/008/330/405/GCF_008330405.1_ASM833040v1
Francisella orientalis	FNO190	SAMN02666529	PRJNA240882	GCA_001042565.2	Complete	186.221	32.3	chromosome:NZ_CP011923.2/CP011923.2	1790	2015-06-29T00:00:00Z		ftp://ftp.ncbi.nlm.nih.gov/genomes/all/GCF/001/042/565/GCF_001042565.2_ASM104256v2
Francisella orientalis	FNO191	SAMN02666528	PRJNA240884	GCA_008330485.1	Complete	186.213	32.3	chromosome:NZ_CP022948.1/CP022948.1	1795	2019-09-09T00:00:00Z		ftp://ftp.ncbi.nlm.nih.gov/genomes/all/GCF/008/330/485/GCF_008330485.1_ASM833048v1
Francisella orientalis	FNO117	SAMN02666525	PRJNA240879	GCA_008330365.1	Complete	186.214	32.3	chromosome:NZ_CP022945.1/CP022945.1	1797	2019-09-09T00:00:00Z		ftp://ftp.ncbi.nlm.nih.gov/genomes/all/GCF/008/330/365/GCF_008330365.1_ASM833036v1

Francisella orientalis	F1	SAMN05990858	PRJNA352676	GCA_001885275.1	Complete	185.433	32.3	chromosome:NZ_CP018051.1/CP018051.1	1797	2016-11-22T00:00:00Z		ftp://ftp.ncbi.nlm.nih.gov/genomes/all/GCF/001/885/275/GCF_001885275.1_ASM188527v1
Francisella orientalis	FNO93	SAMN02666522	PRJNA240872	GCA_008330305.1	Complete	186.233	32.3	chromosome:NZ_CP022942.1/CP022942.1	1798	2019-09-09T00:00:00Z		ftp://ftp.ncbi.nlm.nih.gov/genomes/all/GCF/008/330/305/GCF_008330305.1_ASM833030v1
Francisella orientalis	FNO135	SAMN02666526	PRJNA240880	GCA_008330385.1	Complete	186.215	32.3	chromosome:NZ_CP022946.1/CP022946.1	1799	2019-09-09T00:00:00Z		ftp://ftp.ncbi.nlm.nih.gov/genomes/all/GCF/008/330/385/GCF_008330385.1_ASM833038v1
Francisella orientalis	FNO215	SAMN03354401	PRJNA275872	GCA_008330445.1	Complete	186.243	32.3	chromosome:NZ_CP022950.1/CP022950.1	1799	2019-09-09T00:00:00Z		ftp://ftp.ncbi.nlm.nih.gov/genomes/all/GCF/008/330/445/GCF_008330445.1_ASM833044v1
Francisella orientalis	FNO44	SAMN02666518	PRJNA240800	GCA_008330525.1	Complete	186.237	32.3	chromosome:NZ_CP022939.1/CP022939.1	1800	2019-09-09T00:00:00Z		ftp://ftp.ncbi.nlm.nih.gov/genomes/all/GCF/008/330/525/GCF_008330525.1_ASM833052v1
Francisella orientalis	FNO75	SAMN02666521	PRJNA240871	GCA_008330285.1	Complete	18.622	32.3	chromosome:NZ_CP022941.1/CP022941.1	1800	2019-09-09T00:00:00Z		ftp://ftp.ncbi.nlm.nih.gov/genomes/all/GCF/008/330/285/GCF_008330285.1_ASM833028v1
Francisella orientalis	FNO111	SAMN02666524	PRJNA240875	GCA_008330345.1	Complete	186.228	32.3	chromosome:NZ_CP022944.1/CP022944.1	1802	2019-09-09T00:00:00Z		ftp://ftp.ncbi.nlm.nih.gov/genomes/all/GCF/008/330/345/GCF_00833

												0345.1_ASM833034v1
Francisella orientalis	FNO39	SAMN02666512	PRJNA240795	GCA_008330245.1	Complete	186.255	32.3	chromosome:NZ_CP022938.1/CP022938.1	1803	2019-09-09T00:00:00Z		ftp://ftp.ncbi.nlm.nih.gov/genomes/all/GCF/008/330/245/GCF_008330245.1_ASM833024v1
Francisella orientalis	FNO61	SAMN02666519	PRJNA240802	GCA_008330265.1	Complete	186.235	32.3	chromosome:NZ_CP022940.1/CP022940.1	1804	2019-09-09T00:00:00Z		ftp://ftp.ncbi.nlm.nih.gov/genomes/all/GCF/008/330/265/GCF_008330265.1_ASM833026v1
Francisella orientalis	FNO205	SAMN03354400	PRJNA275871	GCA_008330425.1	Complete	186.234	32.3	chromosome:NZ_CP022949.1/CP022949.1	1804	2019-09-09T00:00:00Z		ftp://ftp.ncbi.nlm.nih.gov/genomes/all/GCF/008/330/425/GCF_008330425.1_ASM833042v1
Francisella orientalis	FNO95	SAMN02666523	PRJNA240873	GCA_008330325.1	Complete	186.234	32.3	chromosome:NZ_CP022943.1/CP022943.1	1805	2019-09-09T00:00:00Z		ftp://ftp.ncbi.nlm.nih.gov/genomes/all/GCF/008/330/325/GCF_008330325.1_ASM833032v1
Francisella orientalis	FNO371	SAMN07503464	PRJNA398204	GCA_008330465.1	Complete	186.225	32.3	chromosome:NZ_CP022953.1/CP022953.1	1806	2019-09-09T00:00:00Z		ftp://ftp.ncbi.nlm.nih.gov/genomes/all/GCF/008/330/465/GCF_008330465.1_ASM833046v1
Francisella orientalis	FNO364	SAMN07503448	PRJNA398203	GCA_008330505.1	Complete	186.231	32.3	chromosome:NZ_CP022952.1/CP022952.1	1808	2019-09-09T00:00:00Z		ftp://ftp.ncbi.nlm.nih.gov/genomes/all/GCF/008/330/505/GCF_008330505.1_ASM833050v1
Francisella orientalis	FNO01	SAMN03354399	PRJNA275870	GCA_001190905.2	Complete	186.244	32.3	chromosome:NZ_CP012153.2/CP012153.2	1810	2015-08-03T00:00:00Z		ftp://ftp.ncbi.nlm.nih.gov/genomes/all/GC

												F/001/190/905/GCF_001190905.2_ASM119090v2
Francisella orientalis	FNO222	SAMN03354402	PRJNA275873	GCA_008330545.1	Complete	18.624	32.3	chromosome:NZ_CP022951.1/CP022951.1	1811	2019-09-09T00:00:00Z		ftp://ftp.ncbi.nlm.nih.gov/genomes/all/GCF/008/330/545/GCF_008330545.1_ASM833054v1
Francisella orientalis FNO12	FNO12	SAMN02469074	PRJNA232116	GCA_001042525.2	Complete	186.221	32.3	chromosome:NZ_CP011921.2/CP011921.2	1805	2015-06-29T00:00:00Z		ftp://ftp.ncbi.nlm.nih.gov/genomes/all/GCF/001/042/525/GCF_001042525.2_ASM104252v2
Francisella orientalis FNO24	FNO24	SAMN02584017	PRJNA234502	GCA_001042545.2	Complete	186.232	32.3	chromosome:NZ_CP011922.2/CP011922.2	1800	2015-06-29T00:00:00Z		ftp://ftp.ncbi.nlm.nih.gov/genomes/all/GCF/001/042/545/GCF_001042545.2_ASM104254v2
Francisella orientalis LADL 07-285A	LADL--07-285A	SAMN02641525	PRJNA210898	GCA_000505725.1	Complete	185.899	32.3	chromosome:NC_023029.1/CP006875.1	1764	2013-12-11T00:00:00Z	ftp://ftp.ncbi.nlm.nih.gov/genomes/all/GCA/000/505/725/GCA_000505725.1_ASM50572v1	ftp://ftp.ncbi.nlm.nih.gov/genomes/all/GCF/000/505/725/GCF_000505725.1_ASM50572v1
Francisella orientalis str. Toba 04	Toba 04	SAMN02604140	PRJNA82619	GCA_000262205.1	Complete	18.472	32.2	chromosome:NC_017909.1/CP003402.1	1786	2012-05-10T00:00:00Z	ftp://ftp.ncbi.nlm.nih.gov/genomes/all/GCA/000/262/205/GCA_000262205.1_ASM26220v1	ftp://ftp.ncbi.nlm.nih.gov/genomes/all/GCF/000/262/205/GCF_000262205.1_ASM26220v1
Francisella persica ATCC VR-331	FSC845	SAMN03777003	PRJNA287166	GCA_001275365.1	Complete	151.668	31.4	chromosome:NZ_CP012505.1/CP012505.1	1221	2015-08-27T00:00:00Z		ftp://ftp.ncbi.nlm.nih.gov/genomes/all/GCF/001/275/365/GCF_001275365.1_ASM127536v1

Francisella persica ATCC VR-331	ATCC VR-331	SAMN00993447	PRJNA73171	GCA_001653955.1	Complete	154.077	31.4	chromosome:NZ_CP013022.1/CP013022.1	1230	2016-06-01T00:00:00Z		ftp://ftp.ncbi.nlm.nih.gov/genomes/all/GCF/001/653/955/GCF_001653955.1_ASM165395v1
Francisella philomiragia	O#319-036 [FSC 153]	SAMN03024268	PRJNA260383	GCA_000833295.1	Complete	192.406	327.977	chromosome:NZ_CP009442.1/CP009442.1; plasmid pFPJ_1:NZ_CP009443.1/CP009443.1	1808	2015-02-06T00:00:00Z	ftp://ftp.ncbi.nlm.nih.gov/genomes/all/GCA/000/833/295/GCA_000833295.1_ASM83329v1	ftp://ftp.ncbi.nlm.nih.gov/genomes/all/GCF/000/833/295/GCF_000833295.1_ASM83329v1
Francisella philomiragia	18844	SAMN16400346	PRJNA668170	GCA_018135955.1	Complete	201.754	325.395	chromosome:NZ_CP063138.1/CP063138.1; plasmid unnamed:NZ_CP063139.1/CP063139.1	1897	2021-04-23T00:00:00Z	ftp://ftp.ncbi.nlm.nih.gov/genomes/all/GCA/018/135/955/GCA_018135955.1_ASM1813595v1	ftp://ftp.ncbi.nlm.nih.gov/genomes/all/GCF/018/135/955/GCF_018135955.1_ASM1813595v1
Francisella philomiragia	O#319-029	SAMN03010442	PRJNA238064	GCA_000833195.1	Complete	204.493	325.919	chromosome:NZ_CP009343.1/CP009343.1; plasmid unnamed:NZ_CP009342.1/CP009342.1	1900	2015-02-06T00:00:00Z	ftp://ftp.ncbi.nlm.nih.gov/genomes/all/GCA/000/833/195/GCA_000833195.1_ASM83319v1	ftp://ftp.ncbi.nlm.nih.gov/genomes/all/GCF/000/833/195/GCF_000833195.1_ASM83319v1
Francisella philomiragia	O#319-067	SAMN03013096	PRJNA259775	GCA_000833215.1	Complete	204.971	325.919	chromosome:NZ_CP009436.1/CP009436.1; plasmid pFPI_1:NZ_CP009437.1/CP009437.1	1915	2015-02-06T00:00:00Z	ftp://ftp.ncbi.nlm.nih.gov/genomes/all/GCA/000/833/215/GCA_000833215.1_ASM83321v1	ftp://ftp.ncbi.nlm.nih.gov/genomes/all/GCF/000/833/215/GCF_000833215.1_ASM83321v1
Francisella philomiragia	GA01-2801	SAMN03023970	PRJNA260288	GCA_000833315.1	Complete	203.371	324.878	chromosome:NZ_CP009444.1/CP009444.1; plasmid pFPK_1:NZ_CP009446.1/CP009446.1; plasmid pFPK_2:NZ_CP009445.1/CP009445.1	1932	2015-02-06T00:00:00Z	ftp://ftp.ncbi.nlm.nih.gov/genomes/all/GCA/000/833/315/GCA_000833315.1_ASM83331v1	ftp://ftp.ncbi.nlm.nih.gov/genomes/all/GCF/000/833/315/GCF_000833315.1_ASM83331v1
Francisella philomiragia	GA01-2794	SAMN03024266	PRJNA260381	GCA_000833255.1	Complete	215.205	323.925	chromosome:NZ_CP009440.1/CP009440.1; plasmid unnamed:NZ_CP00	2024	2015-02-06T00:00:00Z	ftp://ftp.ncbi.nlm.nih.gov/genomes/all/GCA/000/833/2	ftp://ftp.ncbi.nlm.nih.gov/genomes/all/GCF/000/833/25

								9441.1/CP009441.1			55/GCA_000833255.1_ASM83325v1	5/GCF_000833255.1_ASM83325v1
Francisella philomiragia subsp. philomiragia ATCC 25015	O#319L	SAMN03218150	PRJNA239335	GCA_000833455.1	Complete	20.174	32.6	chromosome:NZ_CP010019.1/CP010019.1	1839	2015-02-06T00:00:00Z	ftp://ftp.ncbi.nlm.nih.gov/genomes/all/GCA/000/833/455/GCA_000833455.1_ASM83345v1	ftp://ftp.ncbi.nlm.nih.gov/genomes/all/GCF/000/833/455/GCF_000833455.1_ASM83345v1
Francisella salimarina	CHUGA-F75	SAMN19689720	PRJNA737314	GCA_018972105.1	Complete	194.086	33	chromosome:NZ_CP076680.1/CP076680.1	1837	2021-06-21T00:00:00Z	ftp://ftp.ncbi.nlm.nih.gov/genomes/all/GCA/018/972/105/GCA_018972105.1_ASM1897210v1	ftp://ftp.ncbi.nlm.nih.gov/genomes/all/GCF/018/972/105/GCF_018972105.1_ASM1897210v1
Francisella salina	TX07-7308	SAMN02603541	PRJNA67139	GCA_000219045.1	Complete	203.593	32.9	chromosome:NC_015696.1/CP002872.1	1918	2011-06-20T00:00:00Z	ftp://ftp.ncbi.nlm.nih.gov/genomes/all/GCA/000/219/045/GCA_000219045.1_ASM21904v1	ftp://ftp.ncbi.nlm.nih.gov/genomes/all/GCF/000/219/045/GCF_000219045.1_ASM21904v1
Francisella sp. FSC1006	FSC1006	SAMN03073252	PRJNA261387	GCA_000764555.1	Complete	201.599	32.4	chromosome:NZ_CP009574.1/CP009574.1	1907	2014-10-08T00:00:00Z	ftp://ftp.ncbi.nlm.nih.gov/genomes/all/GCA/000/764/555/GCA_000764555.1_ASM76455v1	ftp://ftp.ncbi.nlm.nih.gov/genomes/all/GCF/000/764/555/GCF_000764555.1_ASM76455v1
Francisella sp. LA112445	LA11-2445	SAMN12027040	PRJNA548429	GCA_012224145.1	Complete	213.967	31.8	chromosome:NZ_CP041030.1/CP041030.1	1994	2020-04-09T00:00:00Z	ftp://ftp.ncbi.nlm.nih.gov/genomes/all/GCA/012/224/145/GCA_012224145.1_ASM1222414v1	ftp://ftp.ncbi.nlm.nih.gov/genomes/all/GCF/012/224/145/GCF_012224145.1_ASM1222414v1
Francisella sp. MA067296	MA067296	SAMN05363344	PRJNA328035	GCA_001879645.1	Complete	182.793	32.494	chromosome:NZ_CP016930.1/CP016930.1; plasmid unnamed1:NZ_CP016929.1/CP016929.1	1683	2016-11-16T00:00:00Z		ftp://ftp.ncbi.nlm.nih.gov/genomes/all/GCF/001/879/645/GCF_001879645.1_ASM187964v1

Francisella sp. Scap27	Scap27	SAMN12071515	PRJNA549111	GCA_013394105.1	Complete	196.729	32.8	chromosome:NZ_CP041326.1/CP041326.1	1828	2020-07-08T00:00:00Z	ftp://ftp.ncbi.nlm.nih.gov/genomes/all/GCA/013/394/105/GCA_013394105.1_ASM1339410v1	ftp://ftp.ncbi.nlm.nih.gov/genomes/all/GCF/013/394/105/GCF_013394105.1_ASM1339410v1
Francisella tularensis	2017314593	SAMN18737171	PRJNA721727	GCA_019134715.1	Complete	189.265	32.3	chromosome:CP073120.1	1434	2021-07-06T00:00:00Z	ftp://ftp.ncbi.nlm.nih.gov/genomes/all/GCA/019/134/715/GCA_019134715.1_ASM1913471v1	
Francisella tularensis	NR-28537	SAMN16400345	PRJNA668347	GCA_014931515.1	Complete	189.019	32.2	chromosome:CP063128.1	1454	2020-10-26T00:00:00Z	ftp://ftp.ncbi.nlm.nih.gov/genomes/all/GCA/014/931/515/GCA_014931515.1_ASM1493151v1	
Francisella tularensis	12T0050_FLI	SAMN08201031	PRJNA422969	GCA_002886065.1	Complete	189.074	32.2	chromosome:CP025778.1	1534	2018-01-17T00:00:00Z		ftp://ftp.ncbi.nlm.nih.gov/genomes/all/GCF/002/886/065/GCF_002886065.1_ASM288606v1
Francisella tularensis	FDAARGOS_598	SAMN10228578	PRJNA231221	GCA_003955795.1	Complete	189.361	32.2	chromosome:NZ_CP034467.1/CP034467.1	1652	2018-12-19T00:00:00Z		ftp://ftp.ncbi.nlm.nih.gov/genomes/all/GCF/003/955/795/GCF_003955795.1_ASM395579v1
Francisella tularensis	FDAARGOS_599	SAMN10228579	PRJNA231221	GCA_003955775.1	Complete	189.285	32.2	chromosome:NZ_CP034466.1/CP034466.1	1663	2018-12-19T00:00:00Z		ftp://ftp.ncbi.nlm.nih.gov/genomes/all/GCF/003/955/775/GCF_003955775.1_ASM395577v1
Francisella tularensis	FDAARGOS_595	SAMN10228575	PRJNA231221	GCA_003955815.1	Complete	189.419	32.1	chromosome:NZ_CP034468.1/CP034468.1	1668	2018-12-19T00:00:00Z		ftp://ftp.ncbi.nlm.nih.gov/genomes/all/GCF/003/955/815/GCF_00395

												5815.1_ASM395581v1
Francisella tularensis	15NIIEG	SAMN04606331	PRJNA317365	GCA_001611815.4	Complete	189.217	32.1	chromosome:NZ_CP066295.1/CP066295.1	1687	2020-12-21T00:00:00Z		ftp://ftp.ncbi.nlm.nih.gov/genomes/all/GCF/001/611/815/GCF_001611815.4_ASM161181v4
Francisella tularensis	NUS 3003ST	SAMN21545791	PRJNA765161	GCA_021560555.1	Complete	189.416	32.2	chromosome:NZ_CP089548.1/CP089548.1	1697	2022-01-24T00:00:00Z	ftp://ftp.ncbi.nlm.nih.gov/genomes/all/GCA/021/560/555/GCA_021560555.1_ASM2156055v1	ftp://ftp.ncbi.nlm.nih.gov/genomes/all/GCF/021/560/555/GCF_021560555.1_ASM2156055v1
Francisella tularensis	2015315990	SAMN18737167	PRJNA721727	GCA_019134815.1	Complete	189.223	32.3	chromosome:NZ_CP073124.1/CP073124.1	1698	2021-07-06T00:00:00Z	ftp://ftp.ncbi.nlm.nih.gov/genomes/all/GCA/019/134/815/GCA_019134815.1_ASM1913481v1	ftp://ftp.ncbi.nlm.nih.gov/genomes/all/GCF/019/134/815/GCF_019134815.1_ASM1913481v1
Francisella tularensis	FAM SR-2	SAMN21545789	PRJNA765161	GCA_021560595.1	Complete	185.816	32.3	chromosome:NZ_CP089550.1/CP089550.1	1760	2022-01-24T00:00:00Z	ftp://ftp.ncbi.nlm.nih.gov/genomes/all/GCA/021/560/595/GCA_021560595.1_ASM2156059v1	ftp://ftp.ncbi.nlm.nih.gov/genomes/all/GCF/021/560/595/GCF_021560595.1_ASM2156059v1
Francisella tularensis	Scherm	SAMN18737164	PRJNA721727	GCA_019182265.1	Complete	185.793	32.3	chromosome:NZ_CP073127.1/CP073127.1	1770	2021-07-06T08:17:00Z	ftp://ftp.ncbi.nlm.nih.gov/genomes/all/GCA/019/182/265/GCA_019182265.1_ASM1918226v1	ftp://ftp.ncbi.nlm.nih.gov/genomes/all/GCF/019/182/265/GCF_019182265.1_ASM1918226v1
Francisella tularensis	2017317779	SAMN18737169	PRJNA721727	GCA_019134735.1	Complete	188.519	32.2	chromosome:NZ_CP073122.1/CP073122.1	1778	2021-07-06T00:00:00Z	ftp://ftp.ncbi.nlm.nih.gov/genomes/all/GCA/019/134/735/GCA_019134735.1_ASM1913473v1	ftp://ftp.ncbi.nlm.nih.gov/genomes/all/GCF/019/134/735/GCF_019134735.1_ASM1913473v1
Francisella tularensis	2014313438	SAMN18737168	PRJNA721727	GCA_019134755.1	Complete	189.098	32.3	chromosome:NZ_CP073123.1/CP073123.1	1788	2021-07-06T00:00:00Z	ftp://ftp.ncbi.nlm.nih.gov/genomes/all/G	ftp://ftp.ncbi.nlm.nih.gov/genomes/all/GC

											CA/019/134/755/GCA_019134755.1_ASM1913475v1	F/019/134/755/GCF_019134755.1_ASM1913475v1
Francisella tularensis	COLL	SAMN18737165	PRJNA721727	GCA_019134835.1	Complete	189.272	32.3	chromosome:NZ_CP073126.1/CP073126.1	1792	2021-07-06T00:00:00Z	ftp://ftp.ncbi.nlm.nih.gov/genomes/all/GCA/019/134/835/GCA_019134835.1_ASM1913483v1	ftp://ftp.ncbi.nlm.nih.gov/genomes/all/GCF/019/134/835/GCF_019134835.1_ASM1913483v1
Francisella tularensis	Schu S4	SAMN18737163	PRJNA721727	GCA_019137555.1	Complete	18.926	32.3	chromosome:NZ_CP073128.1/CP073128.1	1796	2021-07-06T00:00:00Z	ftp://ftp.ncbi.nlm.nih.gov/genomes/all/GCA/019/137/555/GCA_019137555.1_ASM1913755v1	ftp://ftp.ncbi.nlm.nih.gov/genomes/all/GCF/019/137/555/GCF_019137555.1_ASM1913755v1
Francisella tularensis	Schu S4_249	SAMN18737162	PRJNA721727	GCA_019137575.1	Complete	189.278	32.3	chromosome:NZ_CP073129.1/CP073129.1	1807	2021-07-06T00:00:00Z	ftp://ftp.ncbi.nlm.nih.gov/genomes/all/GCA/019/137/575/GCA_019137575.1_ASM1913757v1	ftp://ftp.ncbi.nlm.nih.gov/genomes/all/GCF/019/137/575/GCF_019137575.1_ASM1913757v1
Francisella tularensis	2016320786	SAMN18737170	PRJNA721727	GCA_019134775.1	Complete	189.251	32.3	chromosome:NZ_CP073121.1/CP073121.1	1808	2021-07-06T00:00:00Z	ftp://ftp.ncbi.nlm.nih.gov/genomes/all/GCA/019/134/775/GCA_019134775.1_ASM1913477v1	ftp://ftp.ncbi.nlm.nih.gov/genomes/all/GCF/019/134/775/GCF_019134775.1_ASM1913477v1
Francisella tularensis subsp. holarctica	KU-1	SAMD00232884	PRJDB10003	GCA_014905905.1	Complete	190.783	32.2	chromosome:NZ_AP023460.1/AP023460.1	1632	2020-09-26T00:00:00Z	ftp://ftp.ncbi.nlm.nih.gov/genomes/all/GCA/014/905/905/GCA_014905905.1_ASM1490590v1	ftp://ftp.ncbi.nlm.nih.gov/genomes/all/GCF/014/905/905/GCF_014905905.1_ASM1490590v1
Francisella tularensis subsp. holarctica	NVF1	SAMD00232883	PRJDB10003	GCA_014905885.1	Complete	190.771	32.2	chromosome:NZ_AP023459.1/AP023459.1	1640	2020-09-26T00:00:00Z	ftp://ftp.ncbi.nlm.nih.gov/genomes/all/GCA/014/905/885/GCA_014905885.1_ASM1490588v1	ftp://ftp.ncbi.nlm.nih.gov/genomes/all/GCF/014/905/885/GCF_014905885.1_ASM1490588v1

Francisella tularensis subsp. holarctica	B-8366	SAMN12754422	PRJNA565628	GCA_010232985.1	Complete	189.275	32.2	chromosome:NZ_CP044003.1/CP044003.1	1668	2020-02-06T00:00:00Z	ftp://ftp.ncbi.nlm.nih.gov/genomes/all/GCA/010/232/985/GCA_010232985.1_ASM1023298v1	ftp://ftp.ncbi.nlm.nih.gov/genomes/all/GCF/010/232/985/GCF_010232985.1_ASM1023298v1
Francisella tularensis subsp. holarctica	425	SAMN03251848	PRJNA240117	GCA_000833515.1	Complete	189.419	32.1	chromosome:NZ_CP010289.1/CP010289.1	1674	2015-02-06T00:00:00Z	ftp://ftp.ncbi.nlm.nih.gov/genomes/all/GCA/000/833/515/GCA_000833515.1_ASM83351v1	ftp://ftp.ncbi.nlm.nih.gov/genomes/all/GCF/000/833/515/GCF_000833515.1_ASM83351v1
Francisella tularensis subsp. holarctica	VT68	SAMN03251846	PRJNA240115	GCA_000833495.1	Complete	189.373	32.2	chromosome:NZ_CP010288.1/CP010288.1	1674	2015-02-06T00:00:00Z	ftp://ftp.ncbi.nlm.nih.gov/genomes/all/GCA/000/833/495/GCA_000833495.1_ASM83349v1	ftp://ftp.ncbi.nlm.nih.gov/genomes/all/GCF/000/833/495/GCF_000833495.1_ASM83349v1
Francisella tularensis subsp. holarctica	FSC201	SAMN03773972	PRJNA765161	GCA_021560575.1	Complete	186.288	32.2	chromosome:NZ_CP089549.1/CP089549.1	1674	2022-01-24T00:00:00Z	ftp://ftp.ncbi.nlm.nih.gov/genomes/all/GCA/021/560/575/GCA_021560575.1_ASM2156057v1	ftp://ftp.ncbi.nlm.nih.gov/genomes/all/GCF/021/560/575/GCF_021560575.1_ASM2156057v1
Francisella tularensis subsp. holarctica	FTT_1	SAMN03023737	PRJNA242267	GCA_000833235.1	Complete	189.278	32.2	chromosome:NZ_CP009693.1/CP009693.1	1677	2015-02-06T00:00:00Z	ftp://ftp.ncbi.nlm.nih.gov/genomes/all/GCA/000/833/235/GCA_000833235.1_ASM83323v1	ftp://ftp.ncbi.nlm.nih.gov/genomes/all/GCF/000/833/235/GCF_000833235.1_ASM83323v1
Francisella tularensis subsp. holarctica	B-8367	SAMN12754423	PRJNA565628	GCA_010232785.1	Complete	189.275	32.2	chromosome:NZ_CP044002.1/CP044002.1	1678	2020-02-06T00:00:00Z	ftp://ftp.ncbi.nlm.nih.gov/genomes/all/GCA/010/232/785/GCA_010232785.1_ASM1023278v1	ftp://ftp.ncbi.nlm.nih.gov/genomes/all/GCF/010/232/785/GCF_010232785.1_ASM1023278v1
Francisella tularensis subsp. holarctica	B-8365	SAMN12754421	PRJNA565628	GCA_010233165.1	Complete	189.315	32.1	chromosome:NZ_CP044004.1/CP044004.1	1680	2020-02-06T00:00:00Z	ftp://ftp.ncbi.nlm.nih.gov/genomes/all/GCA/010/233/165/GCA_0102	ftp://ftp.ncbi.nlm.nih.gov/genomes/all/GCF/010/233/165/GCF_01023

											33165.1_ASM1023316v1	3165.1_ASM1023316v1
Francisella tularensis subsp. holarctica	12T0052	SAMN14604395	PRJNA625652	GCA_019469545.1	Complete	189.102	32.2	chromosome:NZ_CP058275.1/CP058275.1	1689	2021-08-09T00:00:00Z	ftp://ftp.ncbi.nlm.nih.gov/genomes/all/GCA/019/469/545/GCA_019469545.1_ASM1946954v1	ftp://ftp.ncbi.nlm.nih.gov/genomes/all/GCF/019/469/545/GCF_019469545.1_ASM1946954v1
Francisella tularensis subsp. holarctica	B-8364	SAMN12754420	PRJNA565628	GCA_010233385.1	Complete	189.264	32.2	chromosome:NZ_CP044005.1/CP044005.1	1694	2020-02-06T00:00:00Z	ftp://ftp.ncbi.nlm.nih.gov/genomes/all/GCA/010/233/385/GCA_010233385.1_ASM1023338v1	ftp://ftp.ncbi.nlm.nih.gov/genomes/all/GCF/010/233/385/GCF_010233385.1_ASM1023338v1
Francisella tularensis subsp. holarctica	2015321842	SAMN18737166	PRJNA721727	GCA_019134795.1	Complete	189.051	32.2	chromosome:NZ_CP073125.1/CP073125.1	1705	2021-07-06T00:00:00Z	ftp://ftp.ncbi.nlm.nih.gov/genomes/all/GCA/019/134/795/GCA_019134795.1_ASM1913479v1	ftp://ftp.ncbi.nlm.nih.gov/genomes/all/GCF/019/134/795/GCF_019134795.1_ASM1913479v1
Francisella tularensis subsp. holarctica	08T0013	SAMN14604387	PRJNA625652	GCA_019469525.1	Complete	189.373	32.2	chromosome:NZ_CP058301.1/CP058301.1	1709	2021-08-09T00:00:00Z	ftp://ftp.ncbi.nlm.nih.gov/genomes/all/GCA/019/469/525/GCA_019469525.1_ASM1946952v1	ftp://ftp.ncbi.nlm.nih.gov/genomes/all/GCF/019/469/525/GCF_019469525.1_ASM1946952v1
Francisella tularensis subsp. holarctica	A-1341	SAMEA5971373	PRJEB33006	GCA_026427395.1	Complete	189.344	32.2	chromosome:NZ_CP098826.1/CP098826.1	1715	2022-11-28T00:00:00Z		ftp://ftp.ncbi.nlm.nih.gov/genomes/all/GCF/026/427/395/GCF_026427395.1_ASM2642739v1
Francisella tularensis subsp. holarctica	A271_1 (FDC408)	SAMN03773882	PRJNA285142	GCA_002102455.2	Complete	189.368	32.2	chromosome:NZ_CP048229.1/CP048229.1	1723	2020-01-29T00:00:00Z		ftp://ftp.ncbi.nlm.nih.gov/genomes/all/GCF/002/102/455/GCF_002102455.2_ASM210245v2
Francisella tularensis	12T0058	SAMN14604396	PRJNA625652	GCA_019469505.1	Complete	189.416	32.2	chromosome:NZ_CP058274.1/CP058274.1	1734	2021-08-09T00:00:00Z	ftp://ftp.ncbi.nlm.nih.gov/genomes/all/G	ftp://ftp.ncbi.nlm.nih.gov/genomes/all/GC

subsp. holarctica											CA/019/469/505/GCA_019469505.1_ASM1946950v1	F/019/469/505/GCF_019469505.1_ASM1946950v1
Francisella tularensis subsp. holarctica F92	F92	SAMN02603045	PRJNA175244	GCA_000313385.1	Complete	188.689	32.2	chromosome:NC_019537.1/CP003932.1	1668	2012-11-19T00:00:00Z	ftp://ftp.ncbi.nlm.nih.gov/genomes/all/GCA/000/313/385/GCA_000313385.1_ASM31338v1	ftp://ftp.ncbi.nlm.nih.gov/genomes/all/GCF/000/313/385/GCF_000313385.1_ASM31338v1
Francisella tularensis subsp. holarctica FSC200	FSC200	SAMN01085703	PRJNA16087	GCA_000168775.2	Complete	189.416	32.2	chromosome:NC_019551.1/CP003862.1	1694	2012-09-28T00:00:00Z	ftp://ftp.ncbi.nlm.nih.gov/genomes/all/GCA/000/168/775/GCA_000168775.2_ASM16877v2	ftp://ftp.ncbi.nlm.nih.gov/genomes/all/GCF/000/168/775/GCF_000168775.2_ASM16877v2
Francisella tularensis subsp. holarctica FTNF002-00	FTNF002-00	SAMN02603037	PRJNA20197	GCA_000017785.1	Complete	189.091	32.2	chromosome:NC_009749.1/CP000803.1	1668	2007-08-23T00:00:00Z	ftp://ftp.ncbi.nlm.nih.gov/genomes/all/GCA/000/017/785/GCA_000017785.1_ASM1778v1	ftp://ftp.ncbi.nlm.nih.gov/genomes/all/GCF/000/017/785/GCF_000017785.1_ASM1778v1
Francisella tularensis subsp. holarctica LVS	LVS	SAMEA3138197	PRJNA16421	GCA_000009245.1	Complete	189.599	32.2	chromosome:NC_007880.1/AM233362.1	1689	2006-03-02T00:00:00Z	ftp://ftp.ncbi.nlm.nih.gov/genomes/all/GCA/000/009/245/GCA_000009245.1_ASM924v1	ftp://ftp.ncbi.nlm.nih.gov/genomes/all/GCF/000/009/245/GCF_000009245.1_ASM924v1
Francisella tularensis subsp. holarctica LVS	LVS	SAMN03010443	PRJNA236485	GCA_000833335.1	Complete	189.218	32.2	chromosome:NZ_CP009694.1/CP009694.1	1690	2015-02-06T00:00:00Z	ftp://ftp.ncbi.nlm.nih.gov/genomes/all/GCA/000/833/335/GCA_000833335.1_ASM83333v1	ftp://ftp.ncbi.nlm.nih.gov/genomes/all/GCF/000/833/335/GCF_000833335.1_ASM83333v1
Francisella tularensis subsp. holarctica OSU18	OSU18	SAMN02641482	PRJNA32025	GCA_000011405.1	Complete	189.573	32.2	chromosome:NC_017463.1/BK006741.1	1654	2008-10-17T00:00:00Z	ftp://ftp.ncbi.nlm.nih.gov/genomes/all/GCA/000/011/405/GCA_000011405.1_ASM1140v1	ftp://ftp.ncbi.nlm.nih.gov/genomes/all/GCF/000/011/405/GCF_000011405.1_ASM1140v1

Francisella tularensis subsp. holarctica OSU18	OSU18	SAMN02641482	PRJNA17265	GCA_000014605.1	Complete	189.573	32.2	chromosome:NC_008369.1/CP000437.1	1662	2006-09-20T00:00:00Z	ftp://ftp.ncbi.nlm.nih.gov/genomes/all/GCA/000/014/605/GCA_000014605.1_ASM1460v1	ftp://ftp.ncbi.nlm.nih.gov/genomes/all/GCF/000/014/605/GCF_000014605.1_ASM1460v1
Francisella tularensis subsp. holarctica PHIT-FT049	PHIT-FT049	SAMN02641556	PRJNA230014	GCA_000524575.1	Complete	188.119	32.2	chromosome:NZ_CP007148.1/CP007148.1	1554	2014-01-30T00:00:00Z	ftp://ftp.ncbi.nlm.nih.gov/genomes/all/GCA/000/524/575/GCA_000524575.1_ASM52457v1	ftp://ftp.ncbi.nlm.nih.gov/genomes/all/GCF/000/524/575/GCF_000524575.1_ASM52457v1
Francisella tularensis subsp. mediasiatica FSC147	FSC147; GIEM 543	SAMN02604345	PRJNA19571	GCA_000018925.1	Complete	189.389	32.3	chromosome:CP000915.1	1406	2008-05-07T00:00:00Z	ftp://ftp.ncbi.nlm.nih.gov/genomes/all/GCA/000/018/925/GCA_000018925.1_ASM1892v1	ftp://ftp.ncbi.nlm.nih.gov/genomes/all/GCF/000/018/925/GCF_000018925.1_ASM1892v1
Francisella tularensis subsp. novicida	AL97-2214	SAMN03107513	PRJNA260088	GCA_001880205.1	Complete	191.646	32.4	chromosome:NZ_CP009653.1/CP009653.1	1763	2016-11-17T00:00:00Z		ftp://ftp.ncbi.nlm.nih.gov/genomes/all/GCF/001/880/205/GCF_001880205.1_ASM188020v1
Francisella tularensis subsp. novicida	AZ06-7470	SAMN03107514	PRJNA260086	GCA_001880245.1	Complete	192.525	324.409	chromosome:NZ_CP009682.1/CP009682.1; plasmid pFNE_1:NZ_CP009683.1/CP009683.1	1789	2016-11-17T00:00:00Z		ftp://ftp.ncbi.nlm.nih.gov/genomes/all/GCF/001/880/245/GCF_001880245.1_ASM188024v1
Francisella tularensis subsp. novicida	TCH2015	SAMN06481648	PRJNA378245	GCA_002952075.1	Complete	200.009	32.4	chromosome:NZ_CP021490.1/CP021490.1	1812	2018-02-12T00:00:00Z		ftp://ftp.ncbi.nlm.nih.gov/genomes/all/GCF/002/952/075/GCF_002952075.1_ASM295207v1
Francisella tularensis subsp. novicida	DPG 3A-IS	SAMN02769653	PRJNA240119	GCA_000834965.1	Complete	204.703	323.139	chromosome:NZ_CP010103.1/CP010103.1; plasmid unnamed:NZ_CP01	1899	2015-02-09T00:00:00Z	ftp://ftp.ncbi.nlm.nih.gov/genomes/all/GCA/000/834/965/GCA_0008	ftp://ftp.ncbi.nlm.nih.gov/genomes/all/GCF/000/834/965/GCF_00083

								0104.1/CP010104.1			34965.1_ASM83496v1	4965.1_ASM83496v1
Francisella tularensis subsp. novicida D9876	D9876	SAMN03092276	PRJNA235892	GCA_000833355.1	Complete	187.021	32.5	chromosome:NZ_CP009607.1/CP009607.1	1734	2015-02-06T00:00:00Z	ftp://ftp.ncbi.nlm.nih.gov/genomes/all/GCA/000/833/355/GCA_000833355.1_ASM83335v1	ftp://ftp.ncbi.nlm.nih.gov/genomes/all/GCF/000/833/355/GCF_000833355.1_ASM83335v1
Francisella tularensis subsp. novicida F6168	F6168	SAMN03010444	PRJNA236045	GCA_000833165.1	Complete	192.724	323.996	chromosome:NZ_CP009353.1/CP009353.1; plasmid unnamed:NZ_CP009352.1/CP009352.1	1764	2015-02-06T00:00:00Z	ftp://ftp.ncbi.nlm.nih.gov/genomes/all/GCA/000/833/165/GCA_000833165.1_ASM83316v1	ftp://ftp.ncbi.nlm.nih.gov/genomes/all/GCF/000/833/165/GCF_000833165.1_ASM83316v1
Francisella tularensis subsp. novicida PA10-7858	PA10-7858	SAMN05449121	PRJNA215224	GCA_001865695.1	Complete	197.896	32.4	chromosome:NZ_CP016635.1/CP016635.1	1853	2016-11-03T00:00:00Z		ftp://ftp.ncbi.nlm.nih.gov/genomes/all/GCF/001/865/695/GCF_001865695.1_ASM186569v1
Francisella tularensis subsp. novicida U112	U112	SAMN02604254	PRJNA16088	GCA_000014645.1	Complete	191.003	32.5	chromosome:NC_008601.1/CP000439.1	1762	2006-11-28T00:00:00Z	ftp://ftp.ncbi.nlm.nih.gov/genomes/all/GCA/000/014/645/GCA_000014645.1_ASM1464v1	ftp://ftp.ncbi.nlm.nih.gov/genomes/all/GCF/000/014/645/GCF_000014645.1_ASM1464v1
Francisella tularensis subsp. novicida U112	U112	SAMN03107387	PRJNA236529	GCA_000833375.1	Complete	191.059	32.5	chromosome:NZ_CP009633.1/CP009633.1	1764	2015-02-06T00:00:00Z	ftp://ftp.ncbi.nlm.nih.gov/genomes/all/GCA/000/833/375/GCA_000833375.1_ASM83337v1	ftp://ftp.ncbi.nlm.nih.gov/genomes/all/GCF/000/833/375/GCF_000833375.1_ASM83337v1
Francisella tularensis subsp. tularensis	NIH B-38	SAMN03219196	PRJNA240113	GCA_000833475.1	Complete	186.244	32.3	chromosome:NZ_CP010115.1/CP010115.1	1729	2015-02-06T00:00:00Z	ftp://ftp.ncbi.nlm.nih.gov/genomes/all/GCA/000/833/475/GCA_000833475.1_ASM83347v1	ftp://ftp.ncbi.nlm.nih.gov/genomes/all/GCF/000/833/475/GCF_000833475.1_ASM83347v1
Francisella tularensis	WY96	SAMN03817044	PRJNA288604	GCA_001262115.1	Complete	189.814	32.3	chromosome:NZ_CP012037.1/CP012037.1	1776	2015-08-06T00:00:00Z		ftp://ftp.ncbi.nlm.nih.gov/genomes/all/GC

subsp. tularensis												F/001/262/115/GCF_001262115.1_ASM126211v1
Francisella tularensis subsp. tularensis	FSC237	SAMN14604413	PRJNA625652	GCA_019469565.1	Complete	189.278	32.3	chromosome:NZ_CP058276.1/CP058276.1	1800	2021-08-09T00:00:00Z	ftp://ftp.ncbi.nlm.nih.gov/genomes/all/GCA/019/469/565/GCA_019469565.1_ASM1946956v1	ftp://ftp.ncbi.nlm.nih.gov/genomes/all/GCF/019/469/565/GCF_019469565.1_ASM1946956v1
Francisella tularensis subsp. tularensis FSC198	FSC 198	SAMEA3138200	PRJNA17375	GCA_000009325.1	Complete	189.262	32.3	chromosome:NC_008245.1/AM286280.1	1765	2006-07-12T00:00:00Z	ftp://ftp.ncbi.nlm.nih.gov/genomes/all/GCA/000/009/325/GCA_000009325.1_ASM932v1	ftp://ftp.ncbi.nlm.nih.gov/genomes/all/GCF/000/009/325/GCF_000009325.1_ASM932v1
Francisella tularensis subsp. tularensis MA00-2987	MA00-2987	SAMN02595231	PRJNA261847	GCA_001267475.1	Complete	189.264	32.3	chromosome:NZ_CP012372.1/CP012372.1	1773	2015-08-14T00:00:00Z		ftp://ftp.ncbi.nlm.nih.gov/genomes/all/GCF/001/267/475/GCF_001267475.1_ASM126747v1
Francisella tularensis subsp. tularensis NE061598	NE061598	SAMN02604230	PRJNA38289	GCA_000023305.1	Complete	189.268	32.3	chromosome:NC_017453.1/CP001633.1	1762	2009-12-28T00:00:00Z	ftp://ftp.ncbi.nlm.nih.gov/genomes/all/GCA/000/023/305/GCA_000023305.1_ASM2330v1	ftp://ftp.ncbi.nlm.nih.gov/genomes/all/GCF/000/023/305/GCF_000023305.1_ASM2330v1
Francisella tularensis subsp. tularensis SCHU S4	SCHU S4	SAMEA3138185	PRJNA9	GCA_000008985.1	Complete	189.278	32.3	chromosome:NC_006570.2/AJ749949.2	1766	2004-12-14T00:00:00Z	ftp://ftp.ncbi.nlm.nih.gov/genomes/all/GCA/000/008/985/GCA_000008985.1_ASM898v1	ftp://ftp.ncbi.nlm.nih.gov/genomes/all/GCF/000/008/985/GCF_000008985.1_ASM898v1
Francisella tularensis subsp. tularensis SCHU S4	SHU-S4	SAMN03246840	PRJNA239340	GCA_000833535.1	Complete	189.279	32.3	chromosome:NZ_CP010290.1/CP010290.1	1767	2015-02-06T00:00:00Z	ftp://ftp.ncbi.nlm.nih.gov/genomes/all/GCA/000/833/535/GCA_000833535.1_ASM83353v1	ftp://ftp.ncbi.nlm.nih.gov/genomes/all/GCF/000/833/535/GCF_000833535.1_ASM83353v1

Francisella tularensis subsp. tularensis str. SCHU S4 substr. NR-28534	SCHU S4 substr. NR-28534	SAMN03264783	PRJNA270247	GCA_000978785.2	Complete	188.843	322.983	chromosome:NZ_CP010446.2/CP010446.2; plasmid unnamed:NZ_CP010447.1/CP010447.1	1719	2015-04-22T00:00:00Z	ftp://ftp.ncbi.nlm.nih.gov/genomes/all/GCA/000/978/785/GCA_000978785.2_ASM97878v2	ftp://ftp.ncbi.nlm.nih.gov/genomes/all/GCF/000/978/785/GCF_000978785.2_ASM97878v2
Francisella tularensis subsp. tularensis TI0902	TI0902	SAMN02604300	PRJNA64439	GCA_000248435.2	Complete	189.286	32.3	chromosome:NC_016937.2/CP003049.2	1815	2012-02-23T00:00:00Z	ftp://ftp.ncbi.nlm.nih.gov/genomes/all/GCA/000/248/435/GCA_000248435.2_ASM24843v2	ftp://ftp.ncbi.nlm.nih.gov/genomes/all/GCF/000/248/435/GCF_000248435.2_ASM24843v2
Francisella tularensis subsp. tularensis TIGB03	TIGB03	SAMN02604301	PRJNA64441	GCA_000248415.2	Complete	196.861	32.3	chromosome:NC_016933.2/CP003048.2	1866	2012-02-23T00:00:00Z	ftp://ftp.ncbi.nlm.nih.gov/genomes/all/GCA/000/248/415/GCA_000248415.2_ASM24841v2	ftp://ftp.ncbi.nlm.nih.gov/genomes/all/GCF/000/248/415/GCF_000248415.2_ASM24841v2
Francisella tularensis subsp. tularensis WY-00W4114	WY-00W4114	SAMN03266146	PRJNA81045	GCA_001011135.1	Complete	189.925	32.3	chromosome:NZ_CP009753.1/CP009753.1	1757	2015-05-21T00:00:00Z		ftp://ftp.ncbi.nlm.nih.gov/genomes/all/GCF/001/011/135/GCF_001011135.1_ASM101113v1
Francisella tularensis subsp. tularensis WY96-3418	WY96-3418	SAMN02603036	PRJNA18459	GCA_000016105.1	Complete	189.848	32.3	chromosome:NC_009257.1/CP000608.1	1761	2007-03-24T00:00:00Z	ftp://ftp.ncbi.nlm.nih.gov/genomes/all/GCA/000/016/105/GCA_000016105.1_ASM1610v1	ftp://ftp.ncbi.nlm.nih.gov/genomes/all/GCF/000/016/105/GCF_000016105.1_ASM1610v1
Francisella uliginis	TX07-7310	SAMN05464625	PRJNA67141	GCA_001895265.1	Complete	223.738	31.6	chromosome:NZ_CP016796.1/CP016796.1	2019	2016-12-09T00:00:00Z		ftp://ftp.ncbi.nlm.nih.gov/genomes/all/GCF/001/895/265/GCF_001895265.1_ASM189526v1
